# Transcriptional profiling of HERV-K(HML-2) in amyotrophic lateral sclerosis and potential implications for expression of HML-2 proteins

**DOI:** 10.1186/s13024-018-0275-3

**Published:** 2018-08-02

**Authors:** Jens Mayer, Christian Harz, Laura Sanchez, Gavin C. Pereira, Esther Maldener, Sara R. Heras, Lyle W. Ostrow, John Ravits, Ranjan Batra, Eckart Meese, Jose Luis García-Pérez, John L. Goodier

**Affiliations:** 1 Department of Human Genetics, University of Saarland, Homburg, Germany; 2 GENYO. Centre for Genomics and Oncological Research: Pfizer, University of Granada, Andalusian Regional Government, Granada, Spain; 3 McKusick-Nathans Institute of Genetic Medicine, Johns Hopkins University School of Medicine, Baltimore, MD USA; 4 Department of Neurology, Johns Hopkins University School of Medicine, Baltimore, MD 28217 USA; 5 Department of Neurosciences, School of Medicine, UCSD, San Diego, CA USA; 6 MRC Human Genetics Unit, Institute of Genetics and Molecular Medicine (IGMM), University of Edinburgh, Western General Hospital, Edinburgh, UK; 7 Department of Biochemistry and Molecular Biology II, Faculty of Pharmacy, University of Granada, Campus Universitario de Cartuja, 18071 Granada, Spain

**Keywords:** Amyotrophic lateral sclerosis, Human endogenous retrovirus, HERV-K(HML-2), Retrotransposon, Reverse transcription, Provirus, Envelope protein, Gag protein

## Abstract

**Background:**

Amyotrophic lateral sclerosis (ALS) is a fatal neurodegenerative disorder. About 90% of ALS cases are without a known genetic cause. The human endogenous retrovirus multi-copy HERV-K(HML-2) group was recently reported to potentially contribute to neurodegeneration and disease pathogenesis in ALS because of transcriptional upregulation and toxic effects of HML-2 Envelope (Env) protein. Env and other proteins are encoded by some transcriptionally active HML-2 loci. However, more detailed information is required regarding which HML-2 loci are transcribed in ALS, which of their proteins are expressed, and differences between the disease and non-disease states.

**Methods:**

For brain and spinal cord tissue samples from ALS patients and controls, we identified transcribed HML-2 loci by generating and mapping HML-2-specific cDNA sequences. We predicted expression of HML-2 *env* gene-derived proteins based on the observed cDNA sequences. Furthermore, we determined overall HML-2 transcript levels by RT-qPCR and investigated presence of HML-2 Env protein in ALS and control tissue samples by Western blotting.

**Results:**

We identified 24 different transcribed HML-2 loci. Some of those loci are transcribed at relatively high levels. However, significant differences in HML-2 loci transcriptional activities were not seen when comparing ALS and controls. Likewise, overall HML-2 transcript levels, as determined by RT-qPCR, were not significantly different between ALS and controls. Indeed, we were unable to detect full-length HML-2 Env protein in ALS and control tissue samples despite reasonable sensitivity. Rather our analyses suggest that a number of HML-2 protein variants other than full-length Env may potentially be expressed in ALS patients.

**Conclusions:**

Our results expand and refine recent publications on HERV-K(HML-2) and ALS. Some of our results are in conflict with recent findings and call for further specific analyses. Our profiling of HML-2 transcription in ALS opens up the possibility that HML-2 proteins other than canonical full-length Env may have to be considered when studying the role of HML-2 in ALS disease.

**Electronic supplementary material:**

The online version of this article (10.1186/s13024-018-0275-3) contains supplementary material, which is available to authorized users.

## Background

Amyotrophic lateral sclerosis (ALS) is a late-onset fatal neurodegenerative disorder affecting motor neurons and with an incidence of about 2 in 100,000. In the United States of America, approximately 5600 people are diagnosed with ALS each year with an average life expectancy of about two to 5 years from time of diagnosis. The disease is variable, however, and many people live longer. About 90% of ALS cases are sporadic (sALS), the majority with unknown genetic cause, while the remaining cases are familial (fALS). To date at least 25 genes have been linked to ALS [1, 2]. The first ALS gene discovered, superoxide dismutase (*SOD1*) [3], is mutated in about 20% of fALS and up to 4% of sALS cases. *C9orf72* is by far the most frequent gene, accounting for about 35% of fALS and 6% of sALS cases [4]. FUS RNA binding protein (*FUS*) and TAR DNA binding protein (*TDP-43* or *TARDBP*) genes each account for about 4% of fALS cases, but other ALS-associated genes are found in less than 2% of fALS patients. Among the many factors implicated in the development of ALS are certain classes of transposable elements (TEs). TEs include long terminal repeat (LTR) and non-LTR class retrotransposons that move by a “copy and paste” mechanism involving reverse transcription of their RNA and insertion of the cDNA copy at a new genomic location. Among the LTR-retrotransposons, human endogenous retroviruses (HERVs) are remnants of infections by exogenous retroviruses that formed proviruses in the germ line that became inheritable.

Approximately 8% of the human genome mass is directly or indirectly derived from retroviral sequences. HERV sequences are usually several millions of years old and have lost gene-coding competence due to accumulation of nonsense mutations. Although they tend to be transcriptionally repressed, HERV transcripts can be found in many human tissue and cell types, and deregulated transcription of HERV sequences has been reported for a number of human diseases (for reviews, see [5–8]).

The HERV-K(HML-2) group comprises a number of evolutionarily young proviruses, some of which are human-specific or even polymorphic in the human population, suggesting recent insertional activity [9–11]. Several HERV-K(HML-2) (henceforth abbreviated as HML-2) proviruses still encode retroviral proteins or modified derivatives caused by mutations. Some HML-2-encoded proteins, especially those derived from the HML-2 *env* gene region, have been reported to impact cellular physiology (reviewed in [12, 13]).

**Fig. 1:**
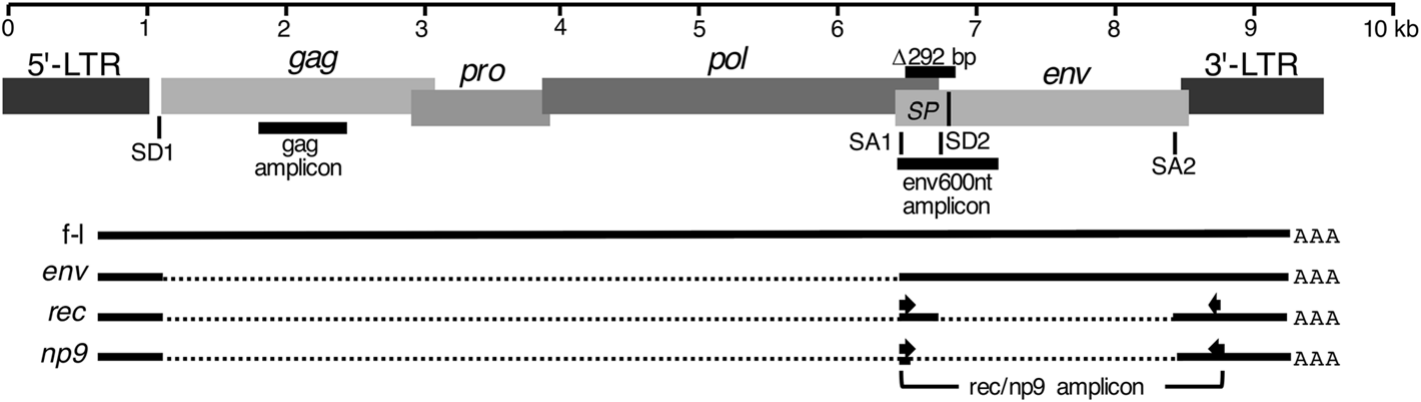
Depiction of a canonical HERV-K(HML-2) type 2 provirus and RT-PCR amplicons. Proviral 5′ and 3′ Long Terminal Repeats (LTR), *gag*, *pro*, *pol* and *env* genes, splice donor (SD) and splice acceptor (SA) sites, splicing patterns of full-length (f-l) proviral transcripts, and three different RT-PCR amplicons for identification of transcribed HML-2 loci within the *gag* and the *env* gene regions (gag amplicon, env600nt amplicon) are indicated, with the *rec*/*np9* amplicon spanning an intron within *env*. Location of PCR primers for amplifcation of *rec*/*np9* transcripts are indicated by arrows. A 292-bp deletion in the *env* gene 5′ region distinguishes HML-2 type 1 from type 2 proviruses and causes an alternative splicing pattern generating the *np9* transcript. Env-SP consisting of the N-terminal 96 aa of full-length Env protein is indicated (see also Fig. 4)

There are two different HML-2 provirus types in the human genome that differ by a 292-bp indel sequence in the *env* gene 5′ region; HML-2 type 1 proviruses lack these 292 bp, while type 2 proviruses contain them (Fig. 1). Type 1 and type 2 proviruses undergo different secondary splicing following initial splicing of *env* mRNA from full-length proviral transcripts. Type 2 proviruses harbor a splice donor (SD) site located within the 292-bp sequence that, in combination with a splice acceptor (SA) just upstream of the proviral 3′ LTR, generates the *rec* transcript lacking most of the (intronic) *env* gene. Type 1 proviruses display an alternative splicing pattern, generating the *np9* transcript by utilizing another SD site that evolved relatively late during primate evolution [14, 15]. Various HML-2 loci in the human genome possess coding capacity for Rec or Np9 protein [16, 17].

Misregulation of HERV-K(HML-2) transcription has been implicated in the etiology of a number of disease conditions, most notably cancers. HML-2 Rec protein was reported to interact with a number of cellular proteins, specifically promyelocytic zinc finger protein (PLZF), testicular zinc finger protein (TZFP), Staufen-1, and human small glutamine-rich tetratricopeptide repeat containing (hSGT), the latter potentially enhancing activity of the androgen receptor. HML-2 Rec may furthermore interfere with germ cell tumor (GCT) development and may directly contribute to tumorigenesis, as it is known to disturb germ cell development in mice and change testis histology towards a carcinoma*-*like phenotype [18–24]. HML-2 Rec was also reported to directly bind to approximately 1600 different cellular RNAs and to modulate their ribosome occupancy with unknown cellular consequences [25].

HML-2 Np9 protein was reported to also interact with PLZF and with the ligand of Numb protein X (LNX), suggesting a possible role of Np9 in tumorigenesis involving the LNX/Numb/Notch pathway [18, 20]. Np9 protein was furthermore reported to activate ERK, AKT and Notch1 pathways, to upregulate β-catenin, to inhibit or promote growth of leukemic cells according to its repression or overexpression, and to display elevated protein levels in leukemia patients [26]. HML-2 Rec and Np9 protein expression has also been demonstrated in different disease contexts, among them GCT, melanoma [27], and in synovia from rheumatoid arthritis [28]. There are recent reports of additional HML-2 protein variants that are due to alternative splicing of the HML-2 *env* gene region or to variant proviruses that potentially generate chimeric HML-2 Env/Rec/Np9 proteins [17, 29, 30]. However, expression of such proteins in vivo and their potential functions remain to be studied.

HML-2 Env protein expression has also been observed in different human tumor types, among them GCT and melanoma (reviewed in ref. [12]). HML-2 Env appears to have tumorigenic potential. Lemaître et al. [31] reported epithelial to mesenchymal transition following HML-2 Env expression and induction of different transcription factors that affect the MAPK/ERK pathway that is associated with cellular transformation processes. Of note, the cytoplasmic tail of the transmembrane (TM) domain of HML-2 Env appears to play an important role in that context [31]. Reduction of HML-2 *env* transcripts by siRNA was reported to reduce tumorigenic potential of a melanoma cell line [32]. A role for HML-2 Env in tumorigenesis and metastasis of breast cancer cells was also reported [33]. Finally, an immunosuppressive domain within the HML-2 Env-TM region inhibited proliferation of human immune cells, modulated expression of cytokines, and altered expression of other cellular genes [34].

While HML-2 type 2 provirus-encoded Env protein is regarded as the canonical HML-2 Env protein, it was recently established that some HML-2 type 1 proviruses encode an Env-like protein that lacks N-terminal regions, but includes outer membrane (OM) and TM regions and that such HML-2 type 1-encoded Env can undergo post-translational processing [35]. It was furthermore shown that canonical HML-2 type 2 Env encodes a ca. 13 kDa signal peptide, named HML-2 SP, that does not enter the usual degradation pathway but is stable and displays protein function different from HML-2 Rec protein with which it shares a long sequence portion. Because of similarities with the Mouse Mammary Tumor Virus encoded p14/SP_Rem_ protein, it is conceivable that HML-2 SP likewise interacts with nucleophosmin (NPM1 or B23) and thus may interfere with important cellular processes [36].

In addition to cancer, HERV involvement in several neurological and neuropsychiatric conditions has been proposed, including multiple sclerosis, schizophrenia, and HIV-associated dementia (reviewed in ref. [37]). HERV-K(HML-2) has also been implicated in ALS. Increased reverse transcriptase activity from an unknown source is detectable in sera and cerebro-spinal fluids of non-HIV-infected sALS patients [38–41]. In one study, an increase in HERV-K *pol* transcripts deriving from several HML-2 and HML-3 loci was reported in brain tissues of ALS patients but not in Parkinson’s patients or accidental death controls [42]. It should be noted, however, that HERV-K(HML-3) is unlikely to encode an active RT due to the overall mutational load of HML-3 sequences [43]. Also co-amplified HML-3 transcripts may have contributed to measurements of overall HML-2 transcript levels in RT-qPCR experiments of that study.

Mutations in the *TDP-43* gene cause a dominant form of ALS and are involved in about 4% of fALS and 1% of sALS patients. TDP-43 protein aggregation has emerged as a unifying pathological marker of a majority of ALS and frontotemporal lobar degeneration (FTLD) cases [44]. Correlation of *TDP-43* and HERV-K(HML-2) RT and *pol* expression in brain tissues has been reported [42, 45], although that link is controversial. Although Li et al. [46] reported TDP-43 protein bound the HERV-K LTR with an attendant increase in HERV-K transcription and RT activity, Manghera et al. [47] found that wild-type TDP-43 binds the HML-2 LTR without altering transcription, while mutant TDP-43 promotes HML-2 RT protein aggregation resulting in its clearance from astrocytes but not neurons. A recent study by Prudencio et al. [48] found no association between *TDP-43* expression in frontal cortex samples or phosphorylated TDP-43 protein levels in FTLD or ALS/FTLD patients and repetitive element expression.

Recently, a number of experiments have implicated HML-2 Env protein as contributing to ALS neurodegeneration. HML-2 Env was reported to be expressed in cortical and spinal neurons of ALS patients, and Env expression affected neurites phenotypically, while mice transgenic for HML-2 *env* showed progressive motor dysfunction accompanied by other ALS-typical anomalies [46]. In sum, while some HERV-K(HML-2)-derived products have been implicated in ALS, their contribution to disease has not been definitively proven.

Transcriptional activity of HERV-K(HML-2) has been studied by different approaches (for instance, see [30, 49–54]). HML-2 transcripts can be identified in a number of tissue and cell types (see above), and there have been various attempts to identify the actual transcribed HML-2 loci contributing to the HML-2 “transcriptome”. Identification of transcribed HML-2 loci provides crucial information as to (i) the source of deregulated transcription of HML-2 loci when comparing diseased and controls, (ii) the actual protein coding-capacity of transcribed HML-2 loci of possible relevance for a specific disease, and (iii) potentially variant HML-2 proteins that should be considered when studying disease relevance.

To identify transcribed HML-2 loci, we and others have employed a strategy involving generation of HML-2 cDNAs, followed by their PCR-amplification, cloning of PCR products, Sanger DNA sequencing and assignment of these sequences to known HML-2 loci by means of pairwise sequence comparisons to identify the HML-2 loci most likely to have generated the original transcripts. Transcribed HML-2 loci have been identified in various tissue and cell types, including GCT, prostate cancer, brain, ALS, melanoma, and embryonic and pluripotent stem cells [29, 30, 42, 50, 55, 56]. It can be concluded from such studies that tissue and cell types display quite different transcription profiles of specific HML-2 proviruses.

An alternative approach for identification of transcribed HML-2 loci involves assignment of HML-2 cDNA sequences that were generated by next generation sequencing, that is, RNA-Seq. For instance, two studies [25, 49] used RNA-sequencing to identify transcribed HML-2 loci in the teratocarcinoma cell line Tera-1 and human blastocysts, respectively. However, such an approach typically generates short sequence reads and so tends to be problematic when cDNA sequences need to be assigned unambiguously to HML-2 loci that are very similar in sequence [49]. This is especially true when assigning RNA-Seq reads to evolutionarily younger, protein-coding-competent HML-2 loci. Furthermore, RNA-Seq can produce biased results because of the relatively low number of reads deriving from HERVs transcribed from their own promoters, compared to RNA-Seq reads from HERV sequences contained within longer gene promoter-driven mRNA transcripts. Quality long sequence reads, as generated by Sanger-based sequencing, currently appear superior for a more unbiased identification of transcribed HML-2 loci.

Precise knowledge of HML-2 loci transcribed in ALS and normal controls allows conclusions concerning HML-2 proteins potentially expressed in ALS and potentially exerting biological functions. To expand upon previously published reports linking HERV-K(HML-2) with ALS, in the present study we applied our recently optimized strategy [17, 30] for identification of transcribed HML-2 loci to profile transcription of HML-2 loci in various tissue samples from mostly sALS or ALS of unknown etiology and control individuals using three different RT-PCR amplicons within the HML-2 proviral sequence. We examined the protein coding capacity of transcribed HML-2 loci with special emphasis on proteins encoded by the HML-2 *env* gene region. We furthermore examined HML-2 transcript levels by RT-qPCR and expression of HML-2 *env* gene-derived proteins in ALS and control tissue samples by Western blotting. We also assayed by RT-qPCR transcript levels of the HERV-W group that has been linked with other neurological diseases, including schizophrenia and multiple sclerosis [37]. Overall, our study did not detect compelling differences in transcription of HML-2 loci in ALS versus control samples. It does, however, provide potentially useful information for further studies on the relevance of HML-2 transcription in ALS, the role of HML-2 encoded proteins in the ALS context, and HML-2 proteins and variants to be considered in future studies.

## Methods

### RNA extraction from tissue samples and cDNA generation for RT-qPCR

Post-mortem brain and spinal cord frozen tissues were obtained from the University of Maryland Brain and Tissue Bank of the NIH Neurobiobank, The Target ALS Multicenter Postmortem Tissue Core of Johns Hopkins University School of Medicine, and the Department of Neurosciences of the University of California San Diego School of Medicine. Human 2102Ep embryonal carcinoma cells (a gift from P.K. Andrews, University of Sheffield) and human embryonic fibroblasts (HEFs, ATCC) were grown in Dulbecco’s modified Eagle’s medium (DMEM) supplemented with 10% FBS and Pen-Strep. H9 (WA09) human Embryonic Stem Cells (hESCs) [57] were obtained from Wicell (RRID: CVCL_9773) and cultured and passaged as previously described [58].

For RNA extractions for RT-qPCR, all brain tissue and some spinal cord tissues were disrupted and homogenized in 500 μl of Trizol (Invitrogen) using the TissueLyser LT (Qiagen). Briefly, 30 mg of sample were transferred to a 2 ml tube containing 250 μl of Trizol and one 5 mm stainless steel bead. The TissueLyser LT program used was 50 Hz for 1 min. After a spin, the supernatant was collected and another 250 μl were added to the sample to repeat the same procedure. Finally, we combined both fractions and proceeded with RNA purification following the Trizol manufacturer’s instructions. Some spinal cord samples were homogenized in 500 μl of Trizol and 5 zirconium silicate beads using a Benchmark BeadBlaster 24. Following centrifugation, the supernatant was further purified using RNeasy Mini Kit with On-column DNase digestion with the RNase-Free DNase Set (Qiagen).

Next, the RNA was treated with RQ1 RNase-free DNase (Promega) for 30 min, purified with ultrapure phenol:chloroform:isoamyl alcohol mixed at 25:24:1 [v/v/v] (Ambion) and precipitated with 3 vol. ice cold 100% ethanol and 0.1 vol. 3 M sodium acetate pH 5.2. Some spinal cord tissues used for RT-qPCR were homogenized in 500 μl of Trizol and zirconium silicate beads using a Benchmark BeadBlaster 24. Following centrifugation, the supernatant was further purified using an RNeasy Mini Kit with On-column DNase digestion (Qiagen). To assure absence of cross-contaminating genomic DNA, 1 μg of total RNA from all samples was treated again with another round of RNase-free DNase I (Invitrogen) for 15 min.

A high-capacity cDNA Reverse Transcription kit (Applied Biosystems) was used to generate cDNA. We included an internal control (no RT added) that was run in all qPCRs (see below).

RNA integrity numbers (RINs) were determined using an Agilent BioAnalyzer and an Agilent RNA 6000 Nano Kit following the manufacturer’s recommendations. Mean RIN was 6.3 (SD = 2.1; 71% with RIN > 5) (Additional file 1: Table S1, Additional file 2: Figure S9). We attribute lower RIN numbers in some samples to long post-mortem intervals affecting tissue quality, two necessary cycles of freezing-thawing of the RNAs, and rigorous DNase-treatments of RNA that were required to remove residual contaminating genomic DNA, a strategy necessary for our sensitive PCR amplification of multi-copy repeat cDNAs lacking introns.

### RT-PCR of HERV-K(HML-2) transcripts

We generated cDNA following previously established procedures [17, 30]. Briefly, we treated approximately 5.5 μg of total RNA with DNase in a total volume of 55 μl using TURBO DNA-free Kit (Ambion/Life Technologies) following the protocol for rigorous DNase treatment. We synthesized cDNA from DNase-treated total RNA (final concentration ~ 40 ng/μl) using Omniscript RT Kit (Qiagen) and including control reactions with identical RNA concentration yet lacking RT enzyme. We subjected 2 μl of the RT(+) and RT(−) reactions to standard PCR with the following cycling parameters: 5′ 95 °C; 35 cycles of 50″ 95 °C, 50″ 53 °C, 1′ 72 °C; 10′ 72 °C. Amplification of an amplicon located within the HERV-K(HML-2) *gag* gene utilized previously described mixes of 4 different forward primers and 3 different reverse primers at relative ratios as previously described [30].

PCR amplification of *rec* and *np9*-related cDNAs was as described [17].

Amplification of PCR products representing approximately 600 bp of the 5′ region of the HERV-K(HML-2) *env* gene utilized the 4 different forward primers mentioned above for *rec*/*np9* transcripts and the following set of reverse primers: env600ntR1: 5′-ATT TAC CCG TGG CCT GAG TG-3′; env600ntR2: 5′-ATT TAC CCG TGG CCT AAG TG-3′; env600ntR3: 5′-ATT TAC CTG TGG CCT GAG CG-3′; env600ntR4: 5′-ATT TAC CTG TGG CCT GAG AG-3′; env600ntR5: 5′-ATT TTA TCT GTG GCC CGA GTG-3′; env600ntR6: 5′-ATT CAT TTG TGA CCT GAG C-3′ mixed at relative ratios of 5:1:1:1:1:1. Cycling parameters of the PCR were as described for the *gag* amplicon (see above). All PCRs had a total volume of 50 μl each. We analyzed results of RT-PCRs by electrophoresis in 1.5% [*w*/*v*] 1xTAE agarose gels containing 0.4 μg/ml of ethidium bromide. We loaded 5 μl (out of 50 μl total PCR volume) from PCRs with RT(+) reactions and 18 μl out of 50 μl total PCR volume from PCRs with RT(−) reactions onto the gel. Only RNA samples not displaying PCR products in RT(−) control reactions were included for further analysis. The strategy for generation of HERV-K(HML-2) cDNA was unstranded, that is, independent of transcriptional direction of loci.

### cDNA sequencing and assignment to HERV-K(HML-2) loci

RT-PCR products from selected ALS and control samples were cloned into pGEM T-Easy vector (Promega). Clones with inserts were identified by colony-PCR. Plasmid DNAs from randomly selected clones were purified and subjected to Sanger DNA sequencing using a vector-specific T7 primer and Applied Biosystems 3730 DNA-Analyzer (Seq-IT GmbH, Kaiserslautern, Germany). Poor quality sequences were excluded from further analysis.

Assignment of cDNA sequences to HERV-K(HML-2) reference and non-reference loci based on locus-specific nucleotide differences followed previously established procedures that generate a relatively unbiased and reproducible identification of transcribed HERV-K(HML-2) loci and their relative transcript levels ([17, 30], and unpublished results). We mapped cDNA sequences to the human GRCh38/hg38 reference genome sequence as provided by the UCSC Genome Browser [59, 60] as well as to reported non-reference HERV-K(HML-2) sequences HERV-K113 [61], HERV-K111 (GenBank acc. no. GU476554; [62]), the so-called “Venter locus” (GenBank acc. no. ABBA01159463; [17, 63]), and additional polymorphic HERV-K(HML-2) type 1 and type 2 sequences recently reported by Wildschutte et al. (Genbank acc. no. KU054272; KU054255; KU054265; KU054266; ref. [10]). Numbers of nucleotide differences of reference genome and non-reference HML-2 loci are depicted for the various RT-PCR amplicons in Additional file 2: Figures S1-S4. For the sake of convenience, chromosomal locations of reference HERV-K(HML-2) loci are given in this paper for human genome sequence version GRCh37/hg19. Problems assigning some of the cDNA sequences due to too few nucleotide (nt) differences between some of the HERV-K(HML-2) loci are addressed in the text.

### Analysis of transcript levels by RT-qPCR

We analyzed in a blinded fashion transcript levels of HERV-K(HML-2) by RT-qPCR employing combinations of four forward and three reverse PCR primers amplifying an approximately 620-bp region of the HERV-K(HML-2) *gag* gene as previously described with minor modifications [30]. Briefly, qPCR cycling parameters were as follows: initial denaturation for 3 min at 95 °C, 40 cycles of 50 s at 95 °C, 50 s at 53 °C, 1 min at 72 °C, and final elongation for 10 min at 72 °C. Duplicate samples were analyzed in a StepOne Real-Time PCR system (Applied Biosystems) using Go Taq qPCR MasterMix (Promega) and PCR primers at 200 nM each. For all samples, GAPDH was used as an internal normalization control, following the strategy of another study of HML-2 expression associated with ALS [46]. PCR primers used were GAPDH Fwd: 5´-TGC ACC ACC AAC TGC TTA GC-3′ and GAPDH Rev.: 5´-GGC ATG GAC TGT GGT CAT GAG-3′. The run method was as follows: 10 min at 95 °C, 40 cycles of 15 s at 95 °C followed by 60 s at 60 °C. A melting curve was subsequently recorded to confirm the identity of amplified products. We employed the ΔΔCt method [64] to determine relative differences in transcript levels. We defined HERV-K(HML-2) transcript levels determined for H9-human embryonic stem cells (hESCs) as 1, as these cells overexpress HERV-K(HML-2) RNAs [65]. Transcript levels were plotted as “Fold change in transcript level” with respect to the transcript level in H9-hESCs (=1). Standard deviations were calculated based on 4 data points per sample derived from duplicate measurements and a technical replicate for each sample [65, 66]. Scatter plots showed no correlation between age at death or gender of donors and relative HML-2 transcript levels (Additional file 2: Figure S10).

### Protein isolation from tissue samples and western blot

For protein extracts, tissues or cells were lysed in RIPA buffer containing Mammalian Protease Inhibitor Cocktail and PMSF (Sigma) and homogenized with a Diagenode Bioruptor. In the case of tissues, five 2.0 mm diameter Zirconium Silicate Beads (Next Advance) were added to the tubes. Samples were centrifuged at 12,000 rpm at 4 °C for 15 min to recover supernatant. Western blotting and detection of proteins was performed as described [67]. Monoclonal α-Env-TM antibody was from Austral Biologicals (HERM-1811-5). Peroxidase-conjugated secondary antibodies were from Jackson ImmunoResearch Laboratories.

## Results

Recently, Li et al. [46] found elevated expression of HERV-K *gag*, *pol*, and *env* genes in brain tissue samples of ALS patients. Immunostaining detected Env protein in the large pyramidal neurons of the cortex and in the anterior horn neurons of the spinal cord. Overexpression of HML-2 Env protein was reported to exert effects in human neurons as well as in a mouse model leading to motor dysfunction. However, the actual HML-2 loci transcribed in ALS are largely uncharacterized. The identification of transcribed source HML-2 loci can provide important information on deregulated HML-2 loci as well as HML-2 proteins potentially expressed in ALS and normal controls.

To identify expressed HML-2 loci, we employed recently established strategies based on Sanger DNA sequencing of PCR-derived amplicons targeting a region within the *gag* gene, one within the *env* gene, and spliced *rec*/*np9* transcripts [17, 29, 30]. Briefly, the strategy involved specific amplification of HML-2 transcript portions by RT-PCR, followed by cloning of RT-PCR products, random selection of insert-harboring clones, and Sanger-sequencing of selected clones. cDNA sequences generated were then mapped to the human reference genome sequence (build GRCh38/hg38) and non-reference polymorphic HERV-K(HML-2) sequences (see the Methods section). We examined in our transcription profiling a total of 32 bulk tissue samples from motor cortex and occipital lobe brain regions, and cervical or thoracic spinal cord sections of both diseased patients and controls (Additional file 1: Tables S1, S2). In the following, every number given in the text and tables means a unique patient (except 33 and 60 which are each used for two patients, as indicated in Additional file 1: Table S1). We indicated in tables, in the main paper text and in Additional file 1: Table S1 instances where different tissue types were obtained from the same patient. The nomenclature of patient samples is thus “patient number - brain region”.

There is no established nomenclature for HERV loci. Different designations of HERV-K(HML-2) loci can therefore be found in the literature. We designate specific HML-2 loci by combining designations from two established naming systems, one of which is based on the location of HML-2 loci in chromosomal bands [68], the other on approved designations of transcribed HML-2 loci as assigned by the HUGO Gene Nomenclature Committee (HGNC) [69]. For instance, a HML-2 locus designated “7p22.1” by ref. [68] and “ERVK-6” by HGNC [69] is now given as “chr7p22.1_K-6”. HML-2 loci without a HGNC designation are noted as “chromosomal band_K-*” or by their original popular designation.

### Identification of transcribed HML-2 loci based on a *gag* amplicon

We amplified an approximately 620-nt region located within the HML-2 *gag* gene region by RT-PCR, cloned the cDNA, and performed Sanger DNA sequencing of randomly selected insert-harboring plasmid clones [17, 30]. Our profiling of transcribed HML-2 loci based on a *gag* region included 19 samples from 13 ALS patients (11 motor cortex, 6 occipital region, 2 spinal cord) and 9 samples from 7 controls (5 motor cortex, 2 occipital region, 2 spinal cord). Both motor and occipital tissue samples were available from 5 ALS patients and 2 controls (Additional file 1: Table S2). A total of 820 cDNA sequences (522 from ALS patients and 298 from controls) were generated from those samples and assigned to HML-2 loci, with 8 to 51 (average 29) sequences obtained per sample. We identified a total of 20 different HML-2 loci as transcribed based on the *gag*-derived cDNA sequences. Per sample, our profiling strategy identified, on average, 6.8 (minimum 4, maximum 11) HML-2 loci as transcribed. Assuming equal cloning efficiencies, we observed very different relative cloning frequencies of cDNA sequences originating from the various HML-2 loci indicating different transcript levels of those loci in the investigated samples.

Among all samples examined, transcripts from an HML-2 locus in chromosome 3, here named chr3q12.3_K-5, were most often identified, with an average relative cloning frequency of 49.8% (range 13–84.6%). An HML-2 type 1 locus thus contributes a large proportion of HML-2 RNA (see also below). Loci chr3q21.2_K-4 (12.1%; range 0–37.5%), chr7q34_K-15 (10.4%; range 05–32%) and chr19q13.12_K-29 (6.7%; range 0–18.2%) were frequently detected in the tested samples. The remaining 16 HML-2 loci were identified at relatively low cloning frequencies (average 1.3%; range 0–30%) and cDNAs from several of those loci were identified only occasionally in some of the samples examined (Additional file 1: Table S2). We then compared *gag*-derived cDNA sequences separately to recently reported human non-reference HERV-K(HML-2) sequences, specifically the HERV-K111 [62] and polymorphic HML-2 proviruses [10]. None of the *gag*-derived cDNA sequences could be assigned with certainty to those non-reference HML-2 sequences.

**Fig. 2:**
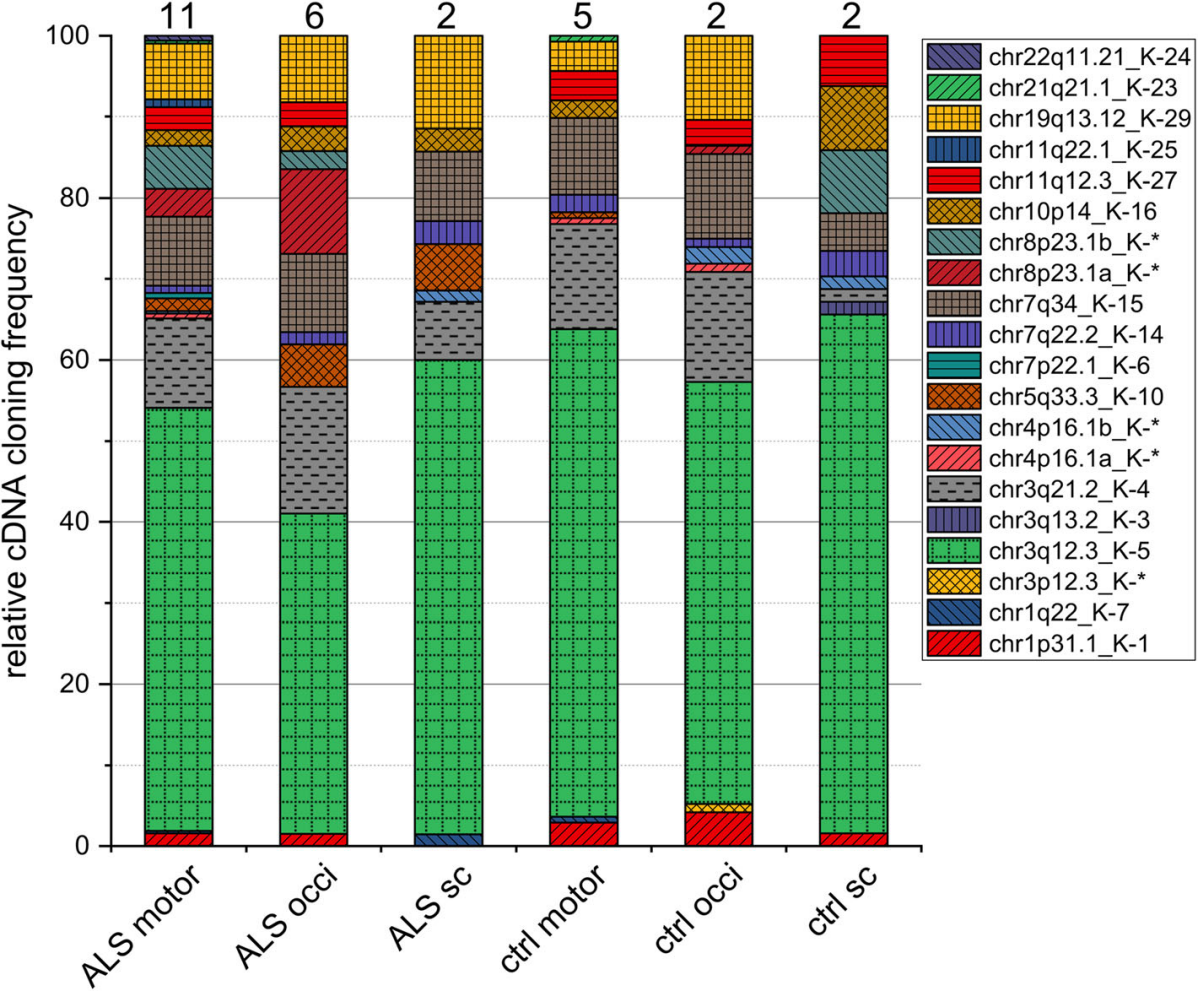
Relative transcript levels of HML-2 loci in ALS and control samples deduced from the *gag* amplicon. Numbers are based on relative cloning frequencies of *gag* amplicon-derived cDNA sequences assignable to different HML-2 loci. Results for tissue samples from motor cortex (motor), occipital cortex (occi) and spinal cord (sc) are each summarized as percentages. Numbers of patients and control donors per tissue type are indicated above each bar. Detailed *gag* amplicon-derived cDNA cloning frequencies for each tissue sample are provided in Additional file 1: Table S2

Notably, we did not obtain strong evidence for HML-2 loci differentially transcribed in ALS disease versus control conditions based on *gag*-derived transcription profiles of HML-2 loci. The four dominantly transcribed HML-2 loci were identified at very similar relative frequencies in both conditions (Table 1, Fig. 2). In detail, when averaging disease versus controls, locus chr3q12.3_K-5 was identified at relative average cloning frequencies of 47.3% in ALS samples versus 55.1% in control samples; locus chr3q21.2_K-4: 12.6% vs. 11.1%; locus chr7q34_K-15: 10.6% vs. 10.1%; locus chr19q13.12_K-29: 7.6% vs. 4.9%, with relatively small ranges between samples (Additional file 1: Table S2). There were only two HML-2 loci identified as transcribed in ALS but not control samples, yet those loci each had relative cDNA cloning frequencies of only 2 out of 522 cDNA sequences for all ALS-derived samples (< 0.4%). The same was true for two HML-2 loci not identified in the ALS state that were each identified by only 1 out of 298 cDNA sequences in control samples.

**Table 1:** Relative cloning frequencies of *gag* amplicon-derived cDNA sequences assignable to unique HERV-K(HML-2) loci^*^

Locus designation	ALS			Ctrl			Type	Env [aa]	Rec/Np9	hg19
	motor	occi	sc	motor	occ	sc				
chr1p31.1_K-1	1.57	1.49		2.90	4.17	1.56	1	606	Np9	chr1:75844857
chr1q22_K-7	0.31		1.43	0.72			1	606	Np9	chr1:155603549
chr3p12.3_K-*					1.04		2	242		chr3:75601670
chr3q12.3_K-5	52.20	39.55	58.57	60.14	52.08	64.06	1	263	Np9	chr3:101412824
chr3q13.2_K-3						1.56	1	434	Np9	chr3:112750203
chr3q21.2_K-4	11.01	15.67	7.14	13.04	13.54	1.56	2		Rec	chr3:125611223
chr4p16.1a_K-*	0.63			0.72	1.04		2	158		chr4:9125672
chr4p16.1b_K-*	0.31		1.43		2.08	1.56	2	342	longer	chr4:9661733
chr5q33.3_K-10	1.57	5.22	5.71	0.72			1	139 (969)		chr5:156091809
chr7p22.1_K-6	0.63						2	699 (f-l)	Rec	chr7:4637944
chr7q22.2_K-14	0.94	1.49	2.86	2.17	1.04	3.13	del			chr7:104392150
chr7q34_K-15	8.49	9.70	8.57	9.42	10.42	4.69	del			chr7:141454695
chr8p23.1a_K-*	3.46	10.45			1.04		2	264		chr8:12081367
chr8p23.1b_K-*	5.35	2.24				7.81	2	264		chr8:12323877
chr10p14_K-16	1.89	2.99	2.86	2.17		7.81	2	286	Rec	chr10:6873520
chr11q12.3_K-27	2.83	2.99		3.62	3.13	6.25	2	337		chr11:62148198
chr11q22.1_K-25	0.94						2	661		chr11:101567880
chr19q13.12_K-29	6.92	8.21	11.43	3.62	10.42		2	206		chr19:37604891
chr21q21.1_K-23	0.31			0.72			1	197	Np9	chr21:19940039
chr22q11.21_K-24	0.63						1	606	Np9	chr22:18934451
# cDNA sequences	318	134	70	138	96	64				
# samples	11	6	2	5	2	2				

^*^Designations of HML-2 loci identified as transcribed are given in the left column. Relative cloning frequencies of cDNA sequences from the HML-2 *gag* amplicon assignable to either of those loci are summarized for motor cortex (motor), occipital region (occi) and spinal cord (sc) tissue samples from patients with ALS and from controls. Total numbers of cDNA sequences assigned for each tissue group and numbers of samples analyzed per tissue group are given in the two bottom lines. Detailed cloning frequencies for each sample are given in Additional file 1: Table S2. For each HML-2 locus, provirus type 1 or 2, the length of the potentially encoded Env protein in number of amino acids (see also Fig. 2) and potential coding capacity for Rec or Np9 proteins are shown in the columns that follow. Note that locus chr7p22.1_K-6 encodes a canonical full-length (f-l) Env protein of 699 aa, and locus chr5q33.3_K-10 potentially encodes an Env protein portion of approximately 139 aa, while the locus also harbors a longer Pro-Env ORF of 969 aa (see text). The rightmost column gives the chromosomal location of the respective HML-2 locus in human reference genome sequence GRCh37/hg19. For the sake of brevity, only one nucleotide position (approximately the central position of the *gag* amplicon) is given. Relative cloning frequencies are also depicted in Fig. 2

Taken together, our *gag*-based analysis revealed a number of transcribed HML-2 loci, some predominantly transcribed, but with overall very similar transcription profiles in ALS-derived samples compared to samples from controls.

### Identification of transcribed HML-2 loci based on cDNA sequences from an *env* gene region

Next, we identified in a complementary approach transcribed HML-2 loci from cDNA sequences derived from the HML-2 *env* gene region. To do so, we designed a PCR amplicon spanning the 292-bp indel region distinguishing type 1 and type 2 HML-2 loci, thus generating PCR products of 330 bp and 620 bp, respectively. Amplicons extended from the canonical start codon of Env, Rec and Np9 downstream into the *env* gene region (Fig. 1). PCR products should thus include both unspliced full-length transcripts and spliced *env* transcripts.

We examined 4 ALS patient samples (3 motor cortex, 1 spinal cord) and 3 control samples (2 motor cortex, 1 spinal cord). PCR products representing *env* gene transcripts from type 1 or type 2 loci were cloned and a total of 144 cDNA sequences were assigned to HML-2 sequences in the human reference genome and to recently reported non-reference HML-2 sequences (Table 2). We assigned, on average, 20.5 (10–40) cDNA sequences per sample. Approximately 96% of cloned cDNA sequences were derived from HML-2 type 1 loci. We note that this skew is likely due to much lower amounts of RT-PCR product from HML-2 type 2 loci (Additional file 2: Figure S8) that subsequently yielded fewer plasmid clones harboring type 2 locus-derived PCR products. Higher amounts of HML-2 type 1 transcript amplified in *env* RT-PCRs are in line with results from the *gag*-based transcription profiling that identified type 1 locus chr3q12.3_K-5 as predominantly transcribed. A total of 93 cDNA sequences were assigned with great confidence to 8 different HML-2 reference loci. Due to insufficient numbers of nucleotide differences, we failed to assign a total of 30 cDNA sequences unambiguously to one of two different HML-2 loci located on chromosome 1 (locus chr1q22_K-7) and chromosome 5 (locus chr5q33.3_K-10). The same applied to 3 cDNA sequences mapping inconclusively to two different HML-2 loci on chromosome 8p23.1 that are located within relatively recently duplicated genome regions and therefore display too few nt differences to permit unambiguous mapping. A total of 18 cDNA sequences likely derived from the so-called “Venter sequence” (GenBank acc. no. ABBA01159463; see ref. [17]), based on one more nt differences in the HERV-K111 sequence (see below).

**Table 2:** Relative cloning frequencies and locus origins of cDNA sequences from an *env* gene 5′ region^*^

locus designation	ALS				Ctrl			Type	Env	Rec/Np9	hg19
	motor	motor	motor	sc	motor	motor	sc				
	89-Mot	62-Mot	64-Mot	802-SC	1509-Mot	73-Mot	4263-SC				
chr1p31.1_K-1				4.00	7.69		4.00	1	606	Np9	chr1:75844857
chr1q23.3_K-18				4.00				1	553		chr1:160667241
chr3q12.3_K-5	62.50	77.50	70.00	28.00	53.85	40.00	40.00	1	263	Np9	chr3:101412824
chr3q13.2_K-3	6.25	2.50			7.69	6.67	4.00	1	434	Np9	chr3:112750203
chr3q21.2_K-4						13.33		2		Rec	chr3:125611223
chr5q33.3_K-10				8.00				1			chr5:156091809
chr11q23.3_K-20						6.67		1	431		chr11:118594283
chr19q11_K-19						6.67		2	699		chr19:28131235
Venter		7.50	10.00	28.00	15.38		20.00	1	452		
chr1:155…/chr5:156…	31.25	12.50	20.00	24.00	15.38	13.33	32.00				
chr8:80…/chr8:12…				4.00		13.33					
# cDNA sequences	16	40	10	25	13	15	25				

^*^A total of 4 samples from ALS patients and 3 samples from controls were examined. A number of cDNA sequences could not be assigned with confidence to either one of two different HML-2 loci on chr1:155,598,877–155,599,165 or chr5:156,087,137–156,087,425, and chr8:8,061,161–8,061,741 or locus chr8:12,318,971–12,319,551. Note that the skew towards type 1 loci is caused by lower amounts of RT-PCR product from type 2 loci and therefore relatively few clones with type 2-derived PCR-product (see the main paper text). See also legends to Tables 1 and 2 for more details

Including the unambiguously assignable cDNA sequences, between 3 and 7 HML-2 loci per sample were identified as transcribed (Table 2). Transcripts very likely deriving from locus chr3q12.3_K-5 were identified in each of the ALS-derived as well as in control samples, with a total of 78 cDNA sequences assignable to that locus, ranging from 28 to 77.5% of cDNA sequences per sample. Also for each sample, between 12.5 and 32% of a total of 30 cDNA sequences were assignable to the chr1q22_K-7/chr5q33.3_K-10 loci.

Transcripts likely originating from locus chr3q13.2_K-3 were identified in 5 out of 7 samples, with relative cloning frequencies up to 7.7%. Transcripts likely originating from the Venter locus (rather than HERV-K111, see above) were also identified in 5 out of 7 samples, with relative cloning frequencies of up to 28%. In addition, transcripts most likely derived from HML-2 loci chr1p31.1_K-1, chr1q23.3_K-18, chr3q21.2_K-4, chr5q33.3_K-10, chr11q23.3_K-20, chr19q11_K-19, and the two inconclusive HML-2 loci in chromosome 8p23.1, were identified less frequently and in fewer samples (Table 2).

In sum, and in agreement with results for the *gag*-based transcription profiling, we did not observe apparent differences when comparing HML-2 loci transcribed in ALS-derived versus control samples.

### Identification of HML-2 loci generating *rec* and *np9* transcripts

Recent studies suggested biological roles for HML-2-encoded Rec and Np9 proteins that are generated from additional splicing events of the HML-2 *env* transcript. Previous studies found *rec* and *np9* transcripts from different HML-2 loci in many human tissue types, including the brain (for instance, see ref. [17]). We therefore examined *rec* and *np9* transcripts present in ALS and control samples using an RT-PCR strategy amplifying larger portions of *rec* and *np9* exons 2 and 3 (Fig. 1), as recently described [17]. RT-PCR products indicative of *rec* and *np9* transcripts could be amplified from all examined ALS and control samples. However, relative amounts of *rec* and *np9* RT-PCR products were variable between samples (Additional file 2: Figure S5).

Further employing our established strategy of identification of HML-2 loci generating *rec* or *np9* transcripts by cloning and sequencing their cDNAs [17], we investigated 5 ALS patient (2 motor cortex, 3 spinal cord) and 2 control samples (1 motor cortex and spinal cord each) and a total of 67 *rec* and 144 *np9* cDNA sequences from these samples (Table 3). Apart from one *rec* cDNA sequence assignable to locus chr3q21.2_K-4 (sample 5458-SC), the remaining 66 cDNA sequences derived from a HML-2 locus in chromosome 5, here named chr5q15_K-31, that represents a retrotransposed *rec* mRNA apparently generated by the LINE-1 (L1) non-LTR retrotransposon machinery in *trans* [17, 70]. Transcription of that HML-2 locus is presumably driven by a flanking non-HML-2 promoter. One out of 67 cDNA sequences isolated from a normal control sample displayed a best match to locus chr3q21.2_K-4.

**Table 3:** Relative cloning frequencies and locus origins of *rec*/*np9* amplicon cDNA sequences^*^

Locus designation	*np9*						*rec*							Type	Rec/Np9	hg19
	ALS					ctrl	ALS					ctrl				
	motor	motor	sc	sc	sc	sc	motor	motor	sc	sc	sc	motor	sc			
	32-Mot	35-Mot	357-SC	5847-SC	802-SC	5458-SC	32-Mot	35-Mot	357-SC	5847-SC	802-SC	23-Mot	5458-SC			
chr3q12.3_K-5	98	5	88	86	15	*33*	0	0	0	0	0	0	0	1	Np9	chr3:101412824
chr3q21.2_K-4	0	16(*)	*0*	*14*	*0*	4(*)	*0*	*0*	*0*	*0*	*0*	*0*	*25*	2	Rec	chr3:125611223
chr5q15_K-31	0	0	0	0	0	0	100	100	100	100	100	100	75	“2”		chr5:92793228
chr22q11.21_K-24	0	0	0	0	8	4	0	0	0	0	0	0	0	1	Np9	chr22:18934451
Venter	0	68	0	0	0	17	0	0	0	0	0	0	0	1		
HERV-K111	2	11	0	0	46	17	0	0	0	0	0	0	0	1		
ambiguous	0	0	12	0	31	25	0	0	0	0	0	0	0			
# cDNA sequences	64	19	17	7	13	24	16	18	2	1	3	23	4			

^*^A total of 7 different ALS-derived and control samples were investigated. Percentages of cDNA sequences assignable to specific HML-2 loci are given. Relative cloning frequencies are separated into *np9* and *rec-*typical lengths. Total numbers of cDNA clones per sample are given in the bottom line. A number of sequences among the np9 cDNA sequences could not be assigned with certainty to specific HML-2 loci in the human reference genome or non-reference HML-2 sequences and were therefore categorized as “ambiguous”. Note that a number of cDNA sequences isolated from sample #35 and assignable to HML-2 type 2 locus chr3q21.2_K-4 appeared as alternatively spliced transcripts of an *np9*-typical length. The same was true for a cDNA sequence isolated from sample 5458-SC and assignable to locus chr3q21.2_K-4. See Table 1 for the three rightmost columns

The HML-2 locus origins of *np9* transcripts were more complex. A total of 5 different HML-2 loci were found to generate *np9* transcripts. Locus chr3q12.3_K-5, a type 1 locus preferentially transcribed based on our *gag* amplicon data (see above), was found to generate *np9* transcripts in all samples examined, yet with quite variable relative cloning frequencies ranging from 5 to 97%. An *np9* transcript from a HML-2 type 1 locus on chromosome 22, chr22q11.21_K-24, was identified at lower relative frequencies in one ALS sample (8%; sample #802-SC) and a control sample (4%; sample #5458-SC) (Table 3).

**Fig. 3:**
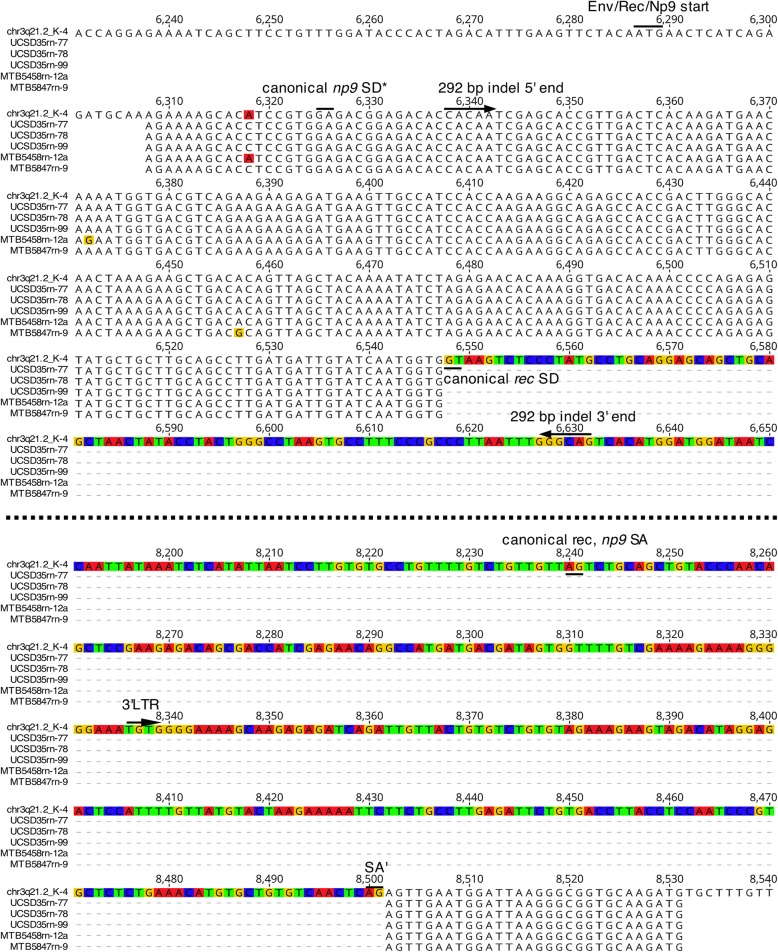
Alternatively spliced transcripts of *np9*-typical length likely generated from HML-2 type 2 locus chr3q21.2_K-4. Five cDNA sequences (with sequences of PCR primers deleted) isolated from 2 different ALS and one control sample are multiply aligned with the sequence of HML-2 locus chr3q21.2_K-4 and with respect to 5′ and 3′ exon sequences within the *env* gene region. Boundaries of the 292-bp region distinguishing HML-2 type 1 and type 2 loci and splice donor (SD) and splice acceptor (SA) sites of canonical *rec* splicing, the Env/Rec/Np9 translational start site, and the 5′ end of the 3’ LTR are indicated. Note that most of the *env* gene (i.e. the *rec* intron 2, see Fig. 1) is omitted from the figure, indicated by the dashed line between the two alignment blocks. Also note that the canonical *np9* SD within the *env* gene is missing in locus chr3q21.2_K-4 (see also ref. [14])

Notably, we also identified a total of 5 *np9*-like transcripts in two ALS and one control samples that most likely were generated not from a type 1 locus but from type 2 locus chr3q21.2_K-4 (second highest relative cloning frequency in the *gag*-based profiling). More detailed analysis of particular cDNA sequences indicated that the *np9*-like transcript from type 2 locus chr3q21.2_K-4 was generated by splicing of an intron (that conformed to the GT-AG rule) beginning from the canonical *rec* SD2 site, but skipping the canonical *rec*/*np9* SA2 site and instead using an alternative site located 260 nt further downstream within the 3′ LTR (Fig. 3).

Several *np9* cDNA sequences did not map to HML-2 loci in the human reference genome but displayed best matches to non-reference HML-2 loci. Specifically, 2, 11 and 46% of *np9* cDNA sequences from three different ALS patient samples (32-Mot; 35-Mot; 802-SC) and 17% of cDNA sequences from a control individual (5458-SC) best matched the recently described HERV-K111 sequence [62]. For those cDNA sequences, intron boundaries within the *env* gene region were in accord with a recent observation that the canonical *np9* SA2 site is skipped due to a AG → GG mutation in the HERV-K111 sequence in favor of another AG located 7 bp downstream [17]. Thus, respective cDNA sequences isolated from three ALS samples and a control sample most likely derived from HERV-K111.

On the other hand, 68 and 17% of *np9* cDNA sequences in one ALS patient sample, (35-Mot) and in a control sample (5458-SC), respectively, best matched the “Venter sequence” that is represented only by a partial HML-2 type 1 locus sequence entry (Genbank acc. no. ABBA01159463). That “Venter sequence” was also identified recently as generating *np9* transcripts in human tissue types including brain [17]. Twelve *np9* cDNA sequences could not be assigned with certainty to HML-2 loci in the human reference sequence or to non-reference HERV-K111 or “Venter” sequences.

Taken together, *rec* and *np9*(−like) transcripts could be identified in ALS and control samples without significant differences. Locus chr5q15_K-31, representing a retrotransposed *rec* mRNA, generated the dominant *rec* transcript in all the investigated samples. A limited number of HML-2 loci generated *np9*-like transcripts with *np9* transcripts from locus chr3q12.3_K-5 being present in all samples. Locus chr22q11.21_K-24 and the non-reference HERV-K111 and “Venter” loci added variable relative numbers of transcripts and comprised the majority of *np9* transcripts in two samples. A cDNA variant of a length similar to that of *np9* was characterized as an alternatively spliced transcript likely produced from HML-2 type 2 locus chr3q21.2_K-4.

### Quantification of HERV-K(HML-2) transcript levels in ALS and control tissues by RT-qPCR

Next, we measured overall levels of HERV-K(HML-2) transcripts by RT-qPCR, employing the above mentioned amplicon within HML-2 *gag*, for a total of 108 ALS and control tissue samples employing previously established strategies [30, 66]. Among these, we assayed 16 cerebellum tissue samples, with 9 of those derived from ALS patients and 7 derived from controls, 30 samples (15 ALS; 15 controls) from thoracic or cervical spinal cord, 35 samples (23 ALS; 12 controls) from motor cortex, 19 samples (14 ALS; 5 controls) from occipital cortex, and 8 samples from hippocampus, all of the latter from ALS patients. For the purpose of comparison, HML-2 transcript levels were also determined for H9 human embryonic stem cells (H9-hESCs) [57], human embryonic fibroblasts (HEFs, obtained from ATCC), and HeLa cervical adenocarcinoma cells. Transcript levels were normalized based on GAPDH transcript levels co-determined for each sample (following the strategy of ref. [46]). Since HERV-K(HML-2) has previously been found to be highly expressed in hESCs, induced pluripotent stem cells, and early human embryogenesis [25, 65], transcript levels determined for H9-hESC cells were furthermore defined as 1 and other transcript levels are given relative to that reference. For comparison with HERV-K(HML-2) levels, we also assayed in all samples transcript levels of HERV-W (Additional file 2: Figure S6) using two previously described primer sets within the HERV-W *env* gene region [71]. HML-2 transcript levels were determined in duplicate for one biological replicate for each brain and spinal cord sample and averaged. There were 4 samples for which only one instead of four data points could be obtained, presumably due to very low HERV-K(HML-2) transcript levels. These were included in our analyses. We considered all measurements of sample-specific transcript levels as real and did not omit possible outliers, and we detected no effect of these on data significance. Furthermore, in light of lower RIN values of some RNAs, we also assessed effects of RNA quality on our analyses by plotting RIN values versus RT-qPCR C_t_-values of GAPDH. Notably, we could not detect a significant effect of RNA quality when the various tissue types were considered separately (Additional file 2: Figure S9). However, a mild effect (R^2^ = 0.38) of RNA quality on C_t_-values was observed when combining RIN and C_t_-values from all samples. A key point is that the omission of samples with lower RINs did not affect our conclusions. In fact, even RNAs with the lowest RIN values produced C_t_-values comparable with RNAs with higher RINs (Additional file 2: Figure S9). See also, for instance, ref. [59] on the validity of samples with reduced RIN values for endpoint RT-PCR and RT-qPCR experiments.

**Fig. 4:**
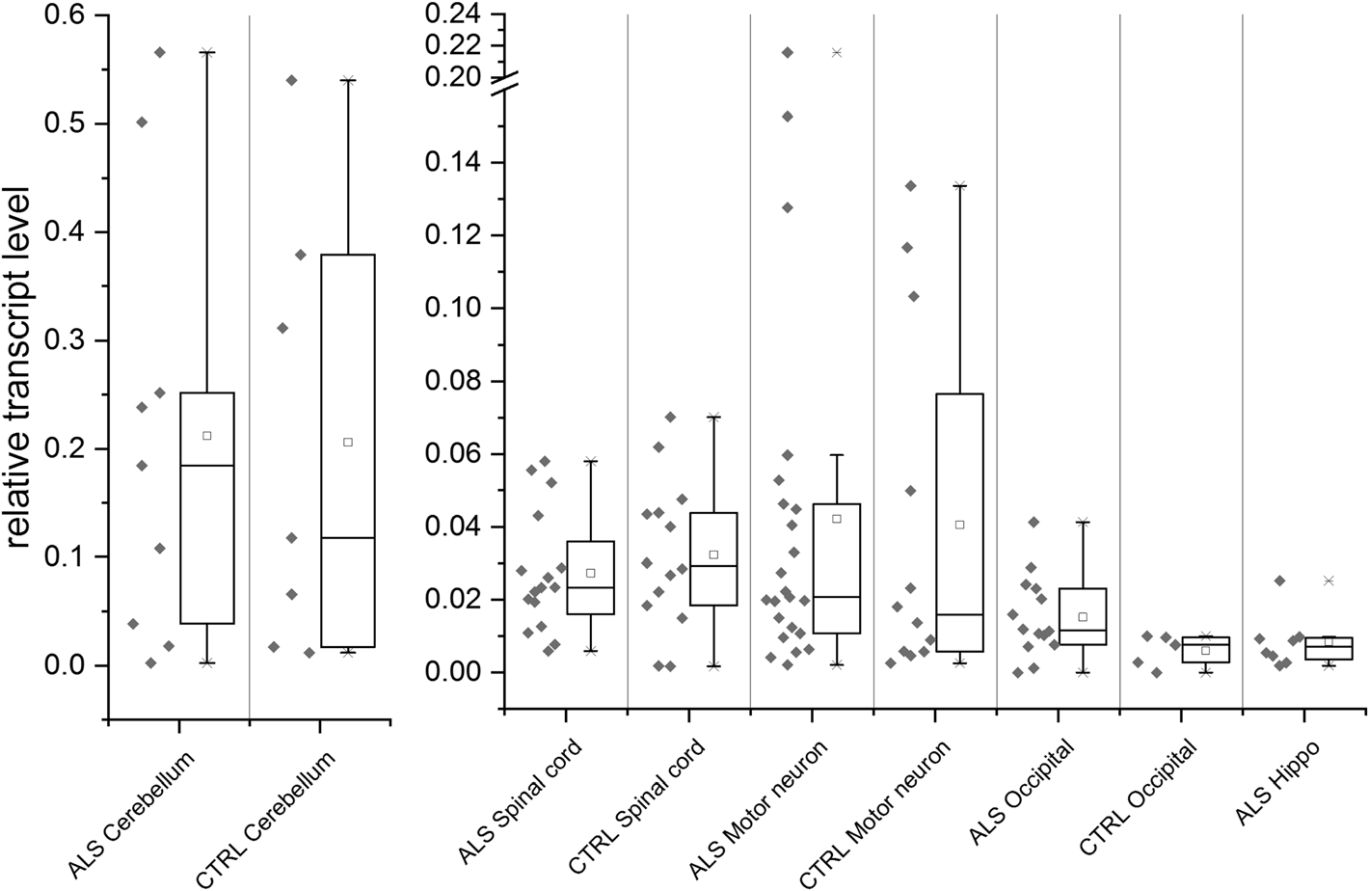
Boxplots of HERV-K(HML-2) overall transcript levels as measured by RT-qPCR. Overall HML-2 transcript levels were measured in samples from various tissue types from ALS patients and controls employing a previously established strategy amplifying a region within the HML-2 *gag* gene [30]. The left boxplots depict normalized transcript levels determined for ALS and control samples from cerebellum. Note the higher relative and much more variable transcript levels in cerebellum. The right boxplots depict normalized HML-2 transcript levels for ALS and control samples from tissue types other than cerebellum. Boxplots depict for each tissue type maximum and minimum values, 95th, 75th, 25th and 5th percentiles, and mean and median, as well as measured transcript levels. Note the break in the Y-axis and that hippocampus included only ALS-derived samples. HML-2 transcript levels are given relative to HML-2 transcript levels co-determined for H9-hESC (set to 1, see text). There were no significantly different transcript levels when comparing ALS and control samples within each tissue type for HML-2 (see the paper text). Significant differential expression was found only for HERV-W transcripts in ALS occipital region samples (see Additional file 2: Figure S6)

Overall, HML-2 transcript levels were considerably different between samples and tissue types (Fig. 4). For instance, average transcript levels ranged between 0.6% for occipital region tissue samples from controls and 21% for ALS-derived cerebellum tissues relative to H9-hESC. When compared to HEFs (not shown in Fig. 4), transcript levels were approximately ten-fold higher in cerebellum, while spinal cord and motor cortex tissues were in the same range and two-fold higher, respectively. Transcript levels for occipital and hippocampal region samples were lower compared to HEFs and rather similar to Hela cells, which were only 0.007% that of H9-hESC (not shown in Fig. 4). HML-2 transcript levels measured for specific samples within tissue types also varied considerably (Fig. 4). For instance, transcript levels in the 30 cortical spinal cord tissue samples ranged from 0.17 to 7%, those in the 35 motor cortex tissue samples from 0.2 to 21.6%, and those in the 16 cerebellum tissue samples from 0.24 to 56% relative to the levels detected in H9-hESC.

More importantly, our analysis did not reveal significantly different transcript levels when comparing ALS and control samples within each tissue type for *p* < 0.05 in a Student’s t-test. A Shapiro-Wilk test did not confirm normal distribution for transcript levels measured in ALS and normal control-derived motor cortex tissue samples. A Wilcoxon-signed-rank test applied to those samples’ transcript levels also established no significant differences (*p* > 0.05) for those tissue types. In the case of HERV-W, while overall transcript levels were much higher compared to HERV-K(HML-2), significantly different transcript levels were detected for the 14 ALS versus 5 control occipital cortex samples only (*p* < 0.04) (Additional file 2: Figure S6). However, further investigations of HERV-W transcription were beyond the scope of this project.

Taken together, our quantification of overall HERV-K(HML-2) transcript levels in more than 100 ALS-derived (40 donors) and control tissue samples (27 donors) revealed considerable variability in transcript levels between samples and tissue types, yet no significant differences in HML-2 transcript levels when comparing ALS-derived and control samples within tissue types.

### *Env* gene-derived proteins potentially encoded by transcribed HERV-K(HML-2) loci

Several HERV-K(HML-2) loci in the human genome are coding-competent for functional proteins. Various proteins encoded by the HML-2 *env* gene region have been identified recently and some of those proteins may interfere with cellular processes (see the Background section). In light of the reported link between HERV-K(HML-2) Env protein expression and motor neuron toxicity [46], we were interested in determining which *env* gene-derived proteins could potentially be encoded by HML-2 loci identified as transcribed by our *gag* or *env* gene-derived cDNA sequencing.

**Fig. 5:**
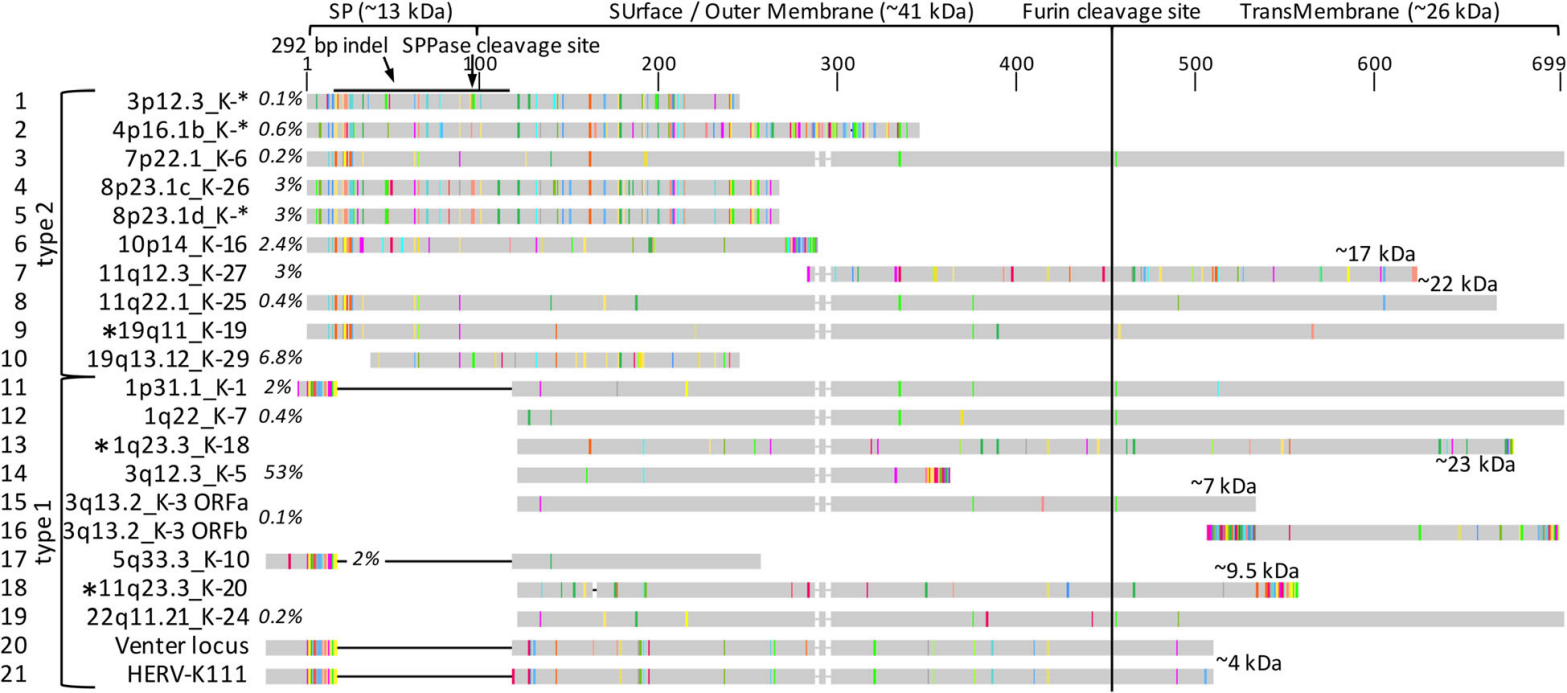
Env(−like) proteins potentially encoded by HERV-K(HML-2) loci identified as transcribed by this study. A full-length, canonical Env protein of 699 aa, as encoded by the HERV-K(HML-2.HOM) type 2 locus, is represented by sequence chr7p22.1_K-6 (=HERV-K(HML-2.HOM); Genbank acc. no.  AF074086; ref. [89]). Grey boxes indicate locations of ORFs within the *env* gene region relative to full-length Env. Amino acid sequence differences among the various sequences are highlighted. Protein sequences as encoded by type 2 and type 1 loci are separated accordingly. The 292-bp indel region distinguishing type 1 and type 2 proviruses on the DNA level is depicted for the topmost sequence. Signal peptide peptidase (SPPase) and furin cleavage sites are annotated and define signal peptide (SP), surface/outer membrane (SU/OM) and transmembrane (TM) regions, if proteins are so processed. Note that the size of the full-length TM domain is around 26 kDa. Predicted protein masses of selected potential TM variants are indicated. Three HML-2 loci, labelled “*” left of sequence names, were identified as transcribed based on a PCR amplicon within the *env* 5′ region. Two different Env partial proteins may be encoded by HML-2 locus 3q13.2_K-3 and are named ORFa and ORFb. HML-2 loci chr1p31.1_K-1, chr5q33.3_K-10, HERV-K111 and the Venter locus harbor longer Pol-Env fusion ORFs extending into upstream proviral regions (see the text). Most of those proteins’ N-terminal portions are omitted from the figure. Relative cloning frequencies of locus-specific cDNAs, as determined by the *gag* amplicon (see Table 1), are given right of the sequence names. Note that locus chr3q12.3_K-5 was found transcribed at the highest relative level (~ 53%)

We first examined the coding competence of transcribed HML-2 loci for Env proteins. Canonical full-length HML-2 type 2 Env protein is 699 amino acids (aa) in length 79 kDa and can be processed into a stable signal peptide (Env-SP) of approx. 13 kDa [36], a surface/outer membrane (SU/OM) domain of approx. 41 kDa, and a transmembrane domain (TM) of approx. 26 kDa due to cleavage by furin endoprotease (see sequence #3 in Fig. 5). Of note, TM protein may run at a higher molecular weight if glycosylated. HML-2 type 1 loci may encode a shorter Env protein variant of approximately 63 kDa lacking N-terminal portions surrounding the 292-bp sequence that is missing in HML-2 type 1 loci, while still including large portions of SU/OM and the TM domains in cases where the open reading frame (ORF) extends downstream into the *env* gene (see #12 and #19 in Fig. 5). Processing of the TM domain has been shown experimentally for such type 1-encoded Env variants [35].

Notably, we found that transcribed HML-2 loci potentially encode a diverse array of Env protein variants (Fig. 5). For instance, type 2 loci chr7p22.1_K-6 and chr19q11_K-19 (sequences #3 and #9 in Fig. 5) potentially encode a canonical Env protein while type 2 locus chr11q22.1_K-25 (#8 in Fig. 5) potentially encodes an Env protein truncated by 38 aa at the C-terminus. Loci 1q22_K-7 (#12, Fig. 5) and chr22q11.21_K-24 (#19, Fig. 5) may encode a “full-length” type 1-like Env protein while type 1 locus chr1q23.3_K-18 (#13, Fig. 5) may encode a similar protein truncated by 28 aa at the C-terminus. HERV-K111 (#20, Fig. 5) and the Venter locus (#21, Fig. 5), both of type 1, may encode Pol-Env fusion proteins reaching just 38 aa into the Env-TM domain. Other HML-2 type 1 and type 2 loci would encode Env(−like) proteins more severely truncated either at the N- or the C-terminus (Fig. 5). A recent ALS-related study [42] reported an HML-2 locus in human chromosome 3q13.2 to harbor an Env ORF. Our own analysis could not confirm such an Env ORF. Rather, this HML-2 type 1 locus, designated chr3q13.2_K-3 in our analysis, harbors a partial internal Env ORF consisting of SU/OM and TM portions, yet lacking 118 aa of its N-terminus and 172 aa of its C-terminus. A second, even shorter ORF comprising approximately 200 aa within Env-TM is also predicted for the chr3q13.2_K-3 locus (#15, #16, Fig. 5).

When taking cloning frequencies of cDNAs from the various HML-2 loci as a rough measure of relative transcript levels, we note that HML-2 loci encoding full-length or near full-length Env proteins, either of type 1 or type 2, appear transcribed at very low levels (#6, #8, #9, #12, #13, #19, Fig. 5) compared with elements containing more truncated open reading frames. Highest relative cDNA cloning frequencies were observed, for example, for locus chr3q12.3_K-5 (53%), potentially translating an internal SU/OM portion of approx. 27 kDa, and locus chr19q13.12_K-29 (6.8%), potentially translating an Env-SP/SU/OM portion of approxately 23 kDa (#14, #10, Fig. 5).

Taken together, a number of Env protein variants comprising internal Env portions and lacking N- or C-terminal portions of variable length may be expressed in ALS and controls. However, canonical full-length HML-2 Env protein is likely expressed at relatively low levels because HML-2 loci capable of generating canonical full-length HML-2 Env protein are very poorly transcribed, at least in samples analyzed for this study.

### Coding-competence of transcribed HML-2 loci for Rec and Np9 proteins

We next were interested in determining if Rec and Np9 proteins could potentially be encoded by HML-2 loci transcribed in our samples. We based our analysis on recent surveys of coding capacities of HML-2 loci for Rec or Np9 proteins, taking into account splice donor and acceptor signals and the presence of ORFs in our mRNA sequences, together with recent findings regarding *rec* and *np9* mRNAs found in various human tissue types [16, 17].

**Fig. 6:**
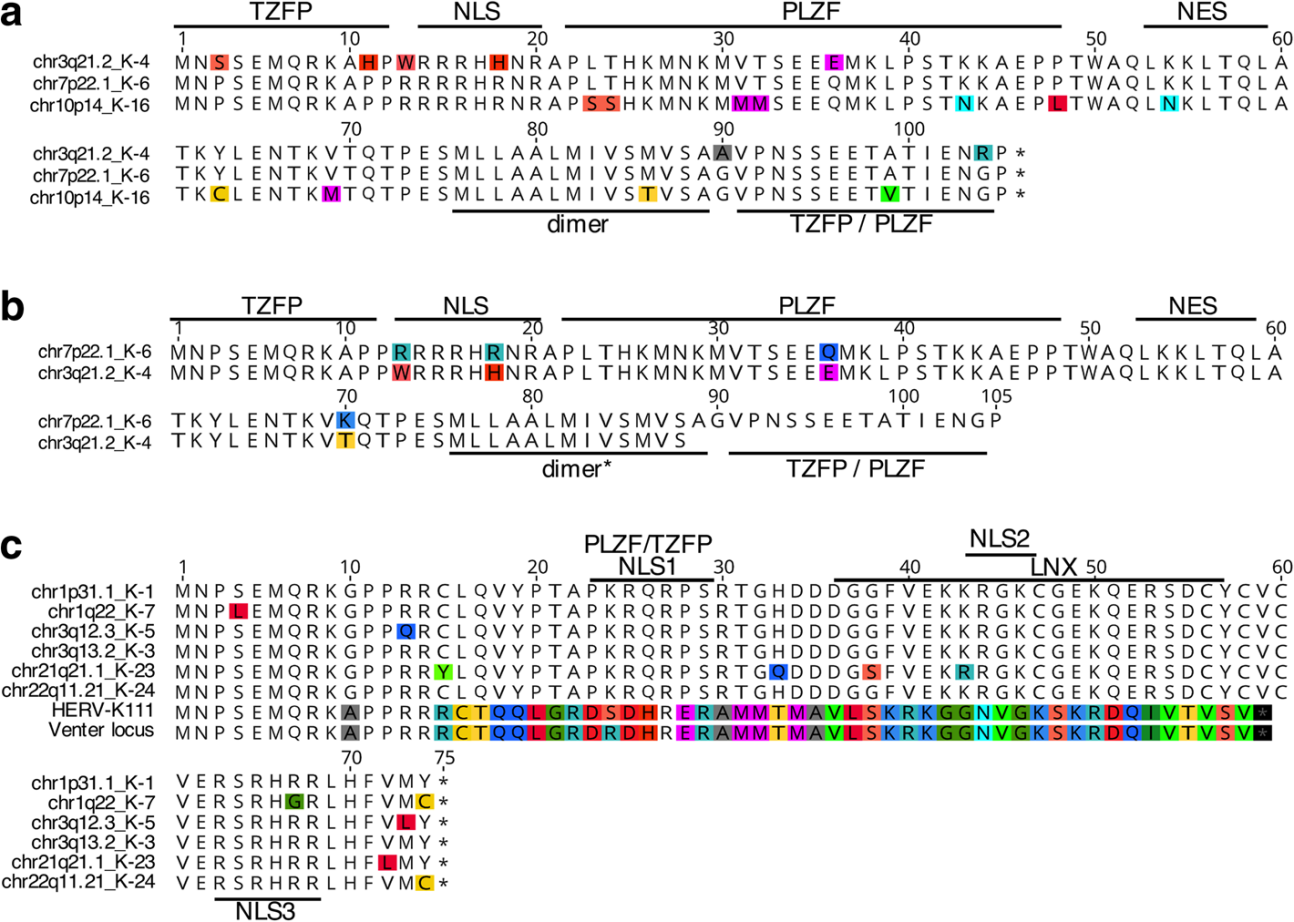
HERV-K(HML-2) Rec and Np9 proteins potentially encoded by transcribed HML-2 loci. Respective amino acid sequences are aligned for (a) Rec and (c) Np9 proteins. Amino acid differences between sequences are highlighted. Previously described domains interacting with other cellular proteins are indicated (see, for instance, ref. [12], and references therein). The chimeric Rec/Np9/Env-like protein potentially encoded by HERV-K111 and the “Venter locus” is depicted in more detail in Fig. 7. **b** A shortened Rec-like protein potentially encoded by alternative *np9*-like splicing of transcripts from HERV-K(HML-2) type 2 locus chr3q21.2_K-4 is compared separately to the Rec protein sequence as encoded by HML-2 locus chr7p22.1_K-6. Note that transcripts from the potentially Rec-encoding locus chr3q21.2_K-4 were identified at an average relative frequency of 10.3% for the *gag* amplicon and less frequently for the *rec*/*np9* and *env* amplicons. Also note that transcripts from the potentially Np9-encoding locus chr3q12.3_K-5 were identified at relative average frequencies of 55% for the *gag*, *rec*/*np9*, and *env* amplicons (see the text and Tables 1–3)

We identified three transcribed HML-2 loci potentially encoding Rec protein, specifically type 2 loci chr3q21.2_K-4 (see also below), chr7p22.1_K-6, and chr10p14_K-16. Relative cDNA cloning frequencies of those three loci were, for all samples combined, 11.3, 0.2 and 2.4%, respectively, and without obvious differences when comparing ALS and control samples. Predicted amino acid (aa) sequences of respective Rec proteins displayed a number of aa differences compared with each other. Interestingly, several of those differences were located within previously identified functional domains of Rec (Fig. 6a).

Our PCR-based approach had identified locus chr5q15_K-31, a retrotransposed *rec* mRNA [17], as predominantly generating *rec*-like transcripts (see above). However, locus chr5q15_K-31 displays a stop codon 19 aa into the Rec encoding region and therefore likely does not encode Rec protein [17].

Another potentially encoded Rec-like protein originating from locus chr3q21.2_K-4 (see above) is likely slightly shortened at the C-terminus due to an alternative, *np9*-like splicing of transcripts (see Fig. 3). This protein would lack the C-terminal 17 aa compared to canonical Rec protein as encoded, for instance, by locus chr7p22.1_K-6 (Fig. 6b). As locus chr3q21.2_K-4 harbors the canonical *rec*/*np9* splice acceptor site, it is unclear whether locus chr3q21.2_K-4 could encode both the canonical length and the shorter Rec proteins.

We furthermore identified six HML-2 loci potentially encoding Np9 protein, specifically loci chr1p31.1_K-1, chr1q22_K-7, chr3q12.3_K-5, chr3q13.2_K-3, chr21q21.1_K-23, and chr22q11.21_K-24 (Fig. 6c). The *gag*-based transcription profiling had produced relative cloning frequencies of 2, 0.4, 53, 0.1, 0.2, and 0.2%, respectively, for those loci. Therefore, locus chr3q12.3_K-5 would be transcribed predominantly compared to other Np9 potentially encoding loci. A higher relative transcript level of locus chr3q12.3_K-5 is also supported by our PCR-mediated approach, which identified *np9* spliced transcripts from locus chr3q12.3_K-5 in all six samples examined, at an average relative cDNA cloning frequency of 54% (Table 3). Furthermore, chr3q12.3_K-5 derived cDNA sequences from the *env* gene 5′ region were frequently seen (53%) (see Table 2). Of further note, Np9 protein encoded by locus chr3q12.3_K-5 does not display aa differences within previously reported Np9 functional domains (Fig. 6b).

It is therefore conceivable that canonical and variant Rec and Np9 proteins capable of exerting previously reported cellular effects are expressed in both ALS and normal control samples.

### Potential expression of chimeric proteins consisting of portions of Env, rec and Np9

Our transcription profiling also identified transcribed HML-2 loci and alternatively spliced transcripts that may encode chimeric proteins consisting of truncated portions of Env, Rec and Np9 proteins.

**Fig. 7:**
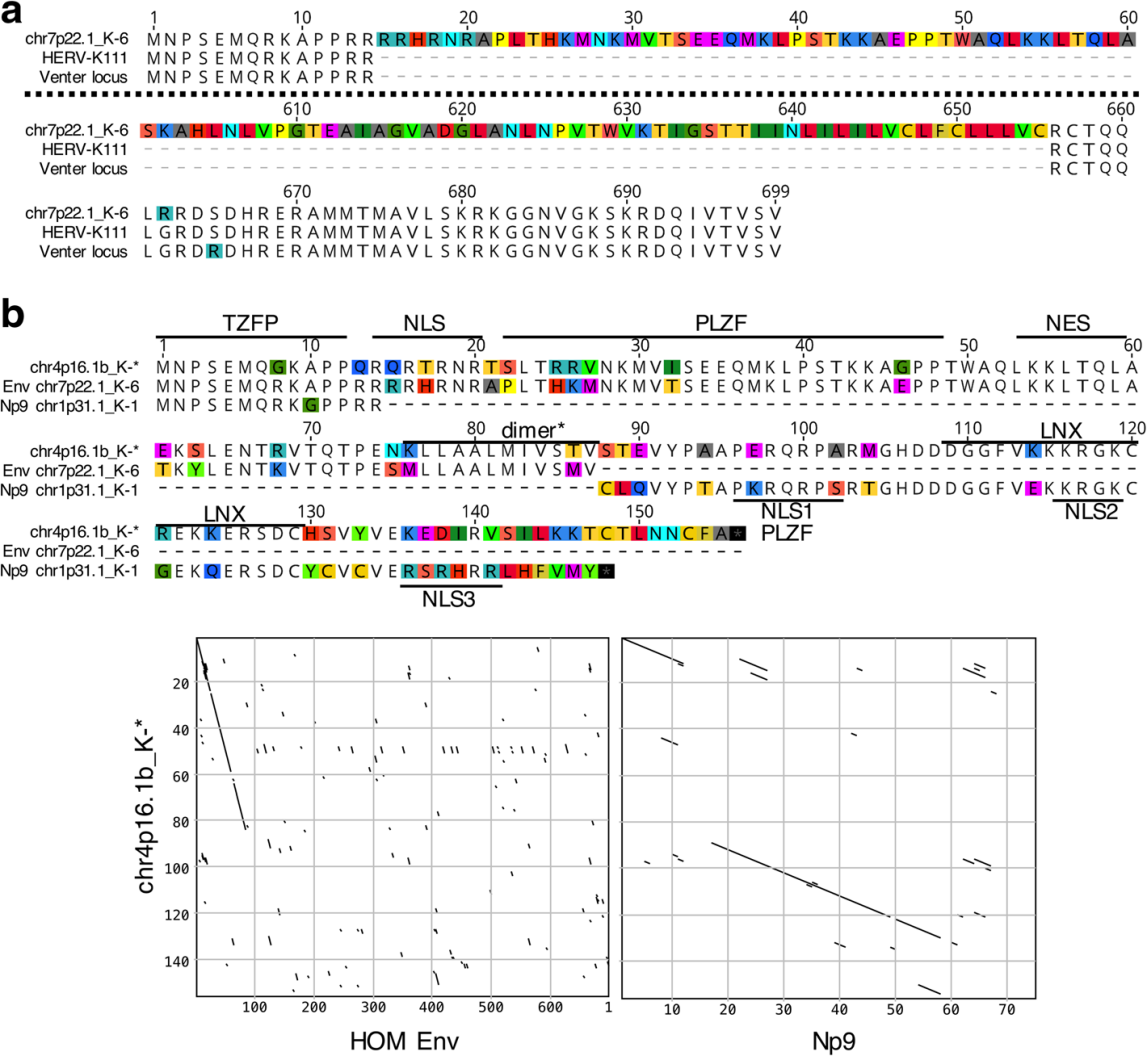
Chimeric Rec/Np9/env proteins potentially encoded by HERV-K111, the Venter locus and locus chr4p16.1b_K-*. **a** Amino acid sequences of proteins potentially encoded by spliced *env* gene transcripts of HERV-K111 and the Venter locus are aligned to the canonical Env protein sequence of locus chr7p22.1_K-6 (=HERV-K(HML-2.HOM); Genbank accession number AF074086; ref. [89]). Note that only the N-terminal 60 aa and the C-terminal 99 aa of chr7p22.1_K-6 Env protein are shown, with both alignment blocks separated by a dashed line. **b** Chimeric Env/Rec/Np9 protein potentially encoded by a spliced transcript from locus chr4p16.1b_K-* multiply aligned with Env/Rec and Np9 protein sequences as encoded by loci chr7p22.1_K-6 (aa 1–87 are shown) and chr1p31.1_K-1. Previously described functional domains within Rec and Np9 are indicated. Differences between protein sequences are highlighted. Dot matrix comparisons below the alignment further depict sequence similarities of respective proteins. Parameters of sequence comparisons were as follows: window size: 5; Min. % score: 50; Hash Value = 1; pam250 scoring matrix

We identified transcripts likely derived from the HERV-K111 and Venter loci, both of which have been identified in previous studies as transcribed [17, 62]. Both loci potentially encode a protein from an *np9*-like spliced transcript that is similar to Env and Rec protein at the N-terminus and to Env protein at the C-terminus, based on sequence comparisons. The chimeric protein would harbor a previously reported TZFP-interacting domain [24] within the Rec-like portion of the protein (Fig. 7a). As up to 46% of cDNA sequences could be assigned to HERV-K111 and up to 68% of cDNA sequences could be assigned to the Venter locus (while some samples examined lacked transcripts assignable to either sequence), it is conceivable that such chimeric protein variants are expressed in some of the ALS and control samples.

We furthermore noted an ORF of 155 aa in length from locus chr4p16.1_K-* that could potentially encode a protein consisting of 87 aa at the N-terminus similar to HML-2 Env, with the remaining protein displaying higher similarities to Np9 protein. Several previously reported functional domains would be present in this chimeric protein but with various aa differences (Fig. 7b). However, we also note that the average relative cloning frequency of transcripts from locus chr4p16.1_K-* was just 0.6%.

### HERV-K(HML-2) Env protein expression in ALS and control tissues

We next used a monoclonal α-Env antibody (HERM-1811-5, Austral Biologicals), recognizing the HML-2 Env C-terminus (for instance, see [72–75]), to assess Env protein expression in ALS and normal spinal cord, motor cortex, cerebellum and frontal cortex samples. The samples and numbers examined for each region are summarized in Additional file 1: Table S1 and representative Western blot results are shown in Additional file 2: Figure S7).

In 2102Ep cells, a human embryonic germ cell teratocarcinoma line that has a transcriptome very similar to hESC [76, 77], this antibody strongly detects a single prominent band of approximately 80 kDa consistent with full-length HERV-K(HML-2) Env protein and occasionally a faint band of about 52 kDa (Additional file 2: Figure S7). A dilution series shows that, using our Western blotting conditions, the antibody is able to detect full-length Env in as little as 6 μg of total cell lysate. Immunofluorescence staining of fixed 2102Ep cells showed full-length HERV-K(HML-2) Env protein to be predominantly cytoplasmic, with some concentration in nucleoli (Additional file 2: Figure S7).

However, full-length Env protein was not detected in any brain or spinal cord samples analyzed, even when 50 μg of whole cell lysate was loaded per well. In a subset of spinal cord and cerebellum samples, 27 kDa bands of varying intensities and consistent in size with the unglycosylated HML-2 Env-TM fragment were seen. In some of these samples, a smaller and fainter 13 kDa band was also occasionally seen. While this size might indicate Env-SP protein, Env-SP and Env-TM protein have no overlapping sequence and could not both be detected by the HERM-1811-5 monoclonal antibody. Previous studies using cell lines have reported that this antibody detects protein species consistent in size with glycosylated Env-TM (32 to 42 kDa) [73–75]. Kammerer et al. [78] reported that this antibody recognized a single epitope, FEASK, within the TM protein. In several of our samples, bands ranging in size from 55 to 65 kDa and of unknown origin were also seen. Contreras-Galindo et al. [72] previously reported that HERM-1811-5 antibody also detects, in NCCIT cells, an approximately 55 kDa band consistent with Env-SU.

In summary, Western blotting, like our RNA analyses, suggests that full-length Env protein is expressed at very low levels in ALS or control brain and spinal cord tissues. However, truncated protein species may bear further investigation when considering a role for HML-2 Env in ALS etiology and progression.

## Discussion

The human endogenous retrovirus group HERV-K(HML-2) has recently been implicated in ALS. Previously, a total of 7 HML-2 loci were identified as preferentially transcribed in ALS patients based on cDNA sequences from an RT-PCR amplicon located within the HML-2 *polymerase* gene [42]. HML-2 Envelope protein was also reported to be expressed in cortical and spinal neurons of ALS patients and to cause motor neuron toxicity following its expression in stem cell-derived human neurons and in a transgenic mouse model that displayed ALS-typical motor neuron and behavioral anomalies. RNA-Seq analysis of several ALS and control samples furthermore identified a great number of HML-2 loci (as many as 54) to be transcribed in ALS and controls. Three HML-2 loci were found transcribed at somewhat higher levels in ALS versus controls, yet with only modest statistical support [46].

As described in the Background section, there is no established optimal strategy for describing transcription of the approximately 70 reference genome and non-reference genome HML-2 loci identified so far [10, 17, 30, 68]. RNA-Seq generating relatively short reads poses considerable detection biases since evolutionarily younger HML-2 are more difficult to identify because of fewer nucleotide differences between loci. Furthermore, a number of HERV sequences are embedded in longer gene transcripts. This may lead to misinterpreting the increased mRNA levels of a gene as autonomous upregulation of its resident HERV sequence. In the case of LINE-1 retrotransposons, for example, Deininger et al. [79] analyzed RNA-Seq data from HeLa cells and found that greater than 99% of LINE-1-related cDNA sequences were within other RNAs and likely not derived from autonomous transcription from an L1 promoter.

Thus, an as accurate as possible identification of transcribed and potentially deregulated HML-2 loci is fundamental to understanding which HML-2 proteins, as well as their actual encoded amino acid sequences, may be expressed in disease versus normal conditions. To contribute to a better understanding of HML-2 transcription and HML-2 proteins potentially expressed in the ALS context, we identified transcribed HML-2 loci by employing previously established Sanger sequencing-based strategies [17, 29, 30] that target three different regions of the HML-2 proviral genome and using primer sets compensating for nt differences between HML-2 loci within respective primer binding regions. Our RT-PCR and Sanger sequencing-based approach identifies transcribed HML-2 loci independent of sense or antisense transcription of respective loci, the latter potentially being due to either antisense promoter activity initiating within the 3′ LTR or a promoter in DNA flanking an HML-2 locus. A recent RNA-Seq-based analysis of HML-2 loci transcribed in the GCT-derived cell line Tera-1, a line that displays a relatively high level of HML-2 transcription, identified some HML-2 loci as antisense transcribed, including loci identified in our present study [49]. Some of those loci appear to generate spliced *rec* or *np9* transcripts, specifically loci 1p31.1_K-1, 1q23.3_K-18, chr3q12.3_K-5, 5p13.3, and 12q14.1_K-21 as revealed here and in a recent study [17]. Because such spliced *rec* and *np9* transcripts can only be generated from HML-2 loci that were transcribed in sense direction it remains to be examined in more detail which HML-2 loci can be transcribed in sense or antisense direction, or both. Analogous with this, human LINE-1 non-LTR retrotransposons also harbor sense and antisense promoters [80].

Our analysis of 32 brain and spinal cord tissue samples from 22 donors identified a total of 24 different HML-2 loci as transcribed. As in previous analyses, there were clearly different relative cloning frequencies of cDNAs from different HML-2 loci: these can be interpreted as different transcript levels (see [29, 30, 49, 50]). For instance, the *gag*-based profiling revealed approximately 53% of all sequences to be derived from locus chr3q12.3_K-5. This locus together with an additional three HML-2 loci comprised approximately 80% of all HML-2 transcripts detected. Thus, only a small number of HML-2 loci predominantly contribute to the HML-2 transcript pool in the ALS and control tissue samples we examined. Similarly, the bulk of LINE-1 mRNAs in cultured cells are generated from a small subset of loci [79, 81].

Similarly, a high relative frequency of HML-2 transcripts from locus chr3q12.3_K-5 was also observed for all examined samples when profiling was based on spliced *rec*/*np9* or *env* amplicons. However, transcript levels for sequences assignable to the Venter locus and to HERV-K111 were more variable between samples, although overall similar numbers of cDNA sequences had been examined per sample. Very similar results were obtained previously when investigating HML-2 locus origins of *rec* and *np9* transcripts in 15 different human normal tissue types [17]. It is currently not clear whether those findings are due to polymorphisms or to greatly variable transcript levels of the HERV-K111 and the Venter loci in our samples. At least 100 sequence variants of HERV-K111 have been reported in human populations [82, 83]. It is conceivable that among our samples several variants are transcribed and that these sequence variants impede unambiguous assignment of cDNA sequences. It is furthermore not clear why HERV-K111 transcripts were identified by *np9*(−like) cDNA sequences but not by the *gag*-based profiling. HERV-K111 was reported to be a full-length type 1 provirus and our PCR primers should have been able to amplify the HERV-K111 *gag* portion. It was also reported that some HERV-K111 variants have 5′ deletions, including portions of *gag* [83]. However, since these HERV-K111 5′ deletions were found in only a minority of the human samples tested, their presence in all our samples seems unlikely.

By mapping RNA-Seq reads to HML-2 loci, a recent study identified HML-2 locus HERV-K(I) to be downregulated 1.09-fold, and loci c7_C and c10_A to be upregulated 1.13-fold and 1.11-fold, respectively, in ALS versus control samples, though with inconclusive statistical support [46]. Those locus designations correspond to HML-2 loci chr3q21.2_K-4, chr7q34_K-15, and chr10p14_K-16, respectively, in our study (for locus designations, see [50, 68, 69, 84]). Likewise, none of those loci showed clear evidence of misregulation in our transcription profilings, just as none of the other HML-2 loci identified as transcribed in our study were consistently altered in disease versus normal states. Our study therefore lends further support to transcription patterns of HML-2 loci being very similar in the ALS versus the control state. This finding might argue against the disease-specific activation of particular, perhaps protein encoding, HML-2 loci playing a causative, direct role in the development of ALS. It is also conceivable that previous observations of differential HERV-K(HML-2) expression levels may relate to altered DNA global methylation status and other epigenetic changes observed in some ALS patients, which might in consequence cause specific HERV-K(HML-2) loci to be differentially transcribed [85–88]. It is furthermore conceivable that differences in cellular composition of brain tissues and cell types actually expressing HERV-K(HML-2) affect analyses. Clearly, future studies using single cell transcriptomics might help to clarify this issue (see also below).

Disease-specific activation of HERV-K(HML-2) locus transcription, as previously reported [42, 46], was likewise not supported by our determination of HERV-K(HML-2) transcript levels. HML-2 transcript levels were also determined for an HML-2 *gag* region in the study by Li et al. [46] and were found to be, on average, 3-fold higher in ALS compared to controls. Of further note, Douville et al. [42] employed an amplicon within the HML-2 *pol* gene and found approximately 2.5-fold higher HML-2 transcript levels in the occipital cortex versus motor cortex, and greatly variable transcript levels in occipital cortex. Our *gag*-based study found HML-2 transcript levels approximately 4-fold higher in motor cortex than occipital cortex and rather limited variable transcript levels in the occipital region. (As a side note, HERV-W transcript levels determined by RT-qPCR were at least 10-fold higher than HERV-K(HML-2) transcript levels when compared with H9-hESCs and at similar levels in occipital and motor cortex tissues.)

Notably, our results are consistent with a recent study that failed to detect global differential expression of either non-LTR or LTR retrotransposon subfamilies in RNA-seq data of sALS vs control brain tissue samples (although significant differences were seen between *C9orf72*-mutant ALS vs sALS patients and controls) [48]. The reasons for discrepancies between our study and other published assessments (for example, ref. [46]) of differential transcription of HML-2 in ALS are unclear. While our sample size was relatively small, we have been able to map transcribed HML-2 cDNA sequences with high confidence. Many HML-2 loci are more or less different in sequence from each other and some of them lack various proviral regions. Therefore, PCR primer sets targeting different HML-2 proviral regions in different RT-qPCR experiments might affect measurements of overall HML-2 transcript levels due to selective exclusion of some HML-2 loci. It is also conceivable that using sets of multiple PCR primers carefully designed to compensate for HML-2 locus-specific nucleotide differences in primer binding regions, as employed in our study, as well as different RT-qPCR conditions, contribute to overall different results. Further refinement of a strategy specifically suited for identification of all transcribed HML-2 loci could yield more conclusive results: information we present here could prove useful in designing such strategies. A strategy capable of determining transcript levels of each HML-2 locus as well as sense and antisense transcription of transcribed HML-2 loci would clearly be favorable. Locus-specific transcriptional activities of HML-2 in different cell types, especially in the ALS context, are currently little investigated (see also below).

The recent study by Li et al. [46] described experimental results in support of a contribution of HML-2 Env protein in ALS. It is conceivable that isoforms other than full-length Env protein were expressed from the *env* gene-containing plasmid constructs used in experiments of that study. *Env* gene transcripts of full-length HML-2 constructs can be efficiently spliced to *rec* transcripts unless splice donor or acceptor sites, and ideally both, are mutated [36]. Since the RT-PCR primers employed by Li et al. would not identify spliced *rec* transcripts and the HERV-K(HML-2) Env antibody raised against Env-TM sequence would not detect Rec protein [46], it is conceivable that Rec protein was (co-)expressed and itself may have affected cellular processes. In this context, Boese et al. [19] found that cellular transformation by HML-2 Env was actually due to Rec protein expressed from a lentiviral vector containing full-length HML-2 *env* that had been spliced to *rec* in the course of the experiment. It is furthermore conceivable that full-length HML-2 Env protein expressed in experiments by Li et al. [46] was processed by signal peptidase to produce a stable Env-SP that may likewise affect cellular processes [36]. An Env-SP would have likewise escaped detection by an antibody directed against HML-2 Env-TM.

We therefore suggest that proteins other than full-length HML-2 Env should be taken into account when considering a causative role of HML-2 in ALS (although we also stress that our study does not provide direct evidence for such an involvement). Our suggestion is supported by our findings that HML-2 loci potentially encoding full-length Env were transcribed only at relatively low levels. Three type 2 loci with (near) full-length Env ORFs (chr7p22.1_K-6, chr19q11_K-19, chr11q22.1_K-25) had cDNA cloning frequencies between 0.2 and 1%. Very low levels of full-length HML-2 Env protein are further supported by our complete failure to detect any full-length HML-2 Env signal by Western blotting of bulk brain or spinal cord tissue lysates of ALS patients or controls, despite very strong signal seen with 2102Ep whole cell lysates having the same total protein concentration.

Similarly, type 1 loci 1q22_K-7 and chr22q11.21_K-24 potentially encoding “full-length” type 1-like Env protein, and type 1 locus chr1q23.3_K-18 potentially encoding a similar protein truncated by 28 aa at its C-terminus, were transcribed at relatively low levels in our samples. Env protein variants from type 1 loci may likewise undergo processing by furin to produce a TM protein of approximately 27 kDa when unmodified, or molecular masses of approximately 32–39 kDa due to glycosylation [35]. Highest relative cDNA cloning frequencies were observed, for example, for locus chr3q12.3_K-5 (53%) potentially translating an internal SU/OM product of approximately 27 kDa, and locus chr19q13.12_K-29 (6.8%) potentially translating an Env-SP/SU/OM product of 23 kDa. All of these protein isoforms would be consistent with the approximately 27 kDa bands detected in our Western blot experiments in a subset of spinal cord and especially cerebellum samples (RT-qPCR results confirmed highest HML-2 *env* transcript levels in cerebellum samples). However, the HERM-1811-5 antibody is directed against HML-2 Env-TM [72, 73, 75, 78]. HML-2 SU/OM and Env-SP/SU/OM proteins thus would remain undetected by this antibody. Our Western blot results therefore suggest that the approximately 27 kDa protein detected in a subset of samples represents Env-TM produced from either an HML-2 type 1 or type 2 *env* gene that furthermore seemed to remain unglycosylated. We failed, however, to associate increased expression of truncated Env protein products with ALS versus control tissue samples.

Rec protein may also be expressed in the ALS context. HML-2 type 2 loci chr3q21.2_K-4 and, to much lesser degree, chr7p22.1_K-6 and chr10p14_K-16 potentially encode Rec proteins. Locus chr3q21.2_K-4 may also generate a Rec-like protein by alternative splicing, shortened by 17 aa and lacking a previously reported TZFP/PLZF binding domain [19, 20, 24]. It is currently not clear how much such aa differences in predicted Rec proteins might alter previously demonstrated protein interactions and biological activities of the Rec variants (see ref. [12], and references therein).

Our analysis also identified several HML-2 loci potentially encoding the Np9 protein. Locus chr3q12.3_K-5 was cloned at high relative levels (~ 53%) in the *gag*-based profiling, and further corroborated by profiling results from the *rec*/*np9* and *env* gene 5′ regions. In light of the previously reported short half-life of Np9 [18], the actual amount of Np9 protein in ALS tissue could be difficult to determine. We note that a transcribed HML-2 locus in human chromosome 22q11.23, not detected as transcribed by our profiling strategy because of mismatches in primer binding sites, potentially encodes an Np9-like protein [29]. It is currently unknown whether this particular locus is also transcribed in the ALS context and whether it should be considered in future studies concerned with HML-2 coding capacity in the ALS context.

Another HML-2 Env protein variant may also have to be considered in the ALS context. We identified in a number of ALS and normal samples relatively high levels of *np9*-like spliced transcript likely originating from the Venter locus and HERV-K111. The predicted protein consists of Env and Rec portions at the N-terminus and an Env portion at the C-terminus. Besides a TZFP-interacting domain in the Env/Rec N-terminal portion, we note that the C-terminal Env portion essentially comprises the cytoplasmic tail of Env-TM that was recently described to be required for activation of the ERK1/2 pathway, including phosphorylation of ERK1/2 and activation of transcription factors *EGR1*, *ETV4* and *ETV5* [31]. The biological relevance for ALS of protein potentially encoded by the Venter locus and HERVK-111 is unknown.

Our study provides potentially valuable insights and hints for future directions in the study of potential HERV-K(HML-2) effects on neurological and neurodegenerative disease. The study also calls for larger scale transcription and proteomic profiling of HML-2 loci in the ALS context in order to better comprehend potential roles of HML-2. Sanger-based DNA sequencing may have advantages over short-read RNA-Seq analyses, which can be confounded by the relatively high sequence similarity of biologically relevant, coding competent HML-2 loci. However, specifically designed strategies for describing HML-2 transcription using high-quality long-read RNA-Seq data would clearly be advantageous considering the much higher amount of sequence information generated compared to Sanger sequencing. Presumably, an optimized RNA-Seq based strategy could also provide much more accurate information on HML-2 locus-specific transcript levels than RT-qPCR alone. On the other side, RNA-Seq requires RNA of higher quality compared to RT-qPCR. Many ALS and control tissue samples may not be suited for RNA-Seq analysis because of too long post-mortem intervals and thus a higher level of RNA degradation. RNA quality may thus pose a main limitation when studying HML-2 expression in the ALS context by RNA-seq. A number of RNA samples in our study had lower RIN values, yet those lower quality RNAs did not significantly affect our results. Instead of RNA-Seq, RT-PCR-based high-throughput amplicon sequencing may be best suited for samples with lower RNA quality.

We further note that possibly a relatively minor proportion of cells in tissue samples from ALS patients may express HML-2 Env protein. In the study of Li et al. [46] only about 20% of cells stained positive by immunofluorescence in frontal cortex and lumbar spinal cord tissue sections when using an Env-TM antibody (and our data suggest it is likely little of that signal derived from the detection of full-length Env protein). The presence of mixed cell type populations in bulk tissue samples could also have an influence on measurements of HML-2 transcript levels: perhaps greater cell type diversity explains the often higher and overall much more variable HML-2 transcript levels we detected in cerebellum versus other tissue types. RNA amplification of bulk tissues may not be the best strategy for determining HML-2 transcript levels in the ALS context. Dilution effects when examining bulk tissues may yield an incomplete depiction of transcribed HML-2 loci and their relative transcript levels in potentially clinically relevant cell types if not sufficiently compensated by a higher number of cDNA sequence reads.

We have been unable to control in our analyses for the selective loss of motor neurons that would be expected to occur in tissues of ALS patients. We note that this is also a potential problem of other studies in this area [42, 46, 48]. Lack of normalization to neuronal loss may currently represent a limitation when studying HML-2 expression in the ALS context. If motor neurons contribute a significant percentage of the HML-2 transcripts in a bulk tissue sample (as suggested in ref. [46]), loss of neurons would reduce apparent overall HML-2 transcript levels in ALS versus control samples. On the other hand, non-neuronal cell types may also contribute HML-2 transcripts at variable levels. Therefore, presorting of bulk neuronal tissues for specific neuronal cell types is one strategy for overcoming this potential bias. Ideally, high-throughput HML-2-specific transcription profiling of single cells sorted by flow cytometry for positive staining with HML-2 Env protein would be useful for identifying HML-2 loci of potential relevance, including the actual HML-2 Env(−like) protein isoforms potentially affecting cellular processes. Such an analysis could be complemented by identification of HML-2 (Env) protein forms actually expressed in respective cell types by immunoprecipitation using isoform-specific antibodies followed by proteomic identification of their sequences.

## Conclusions

HERV-K(HML-2) and HML-2-encoded Envelope protein has recently been implicated in the development of ALS. Our study provides further insight into the relevance of HML-2 for ALS. We found similar transcriptional activities of specific HML-2 loci as well as statistically indifferent overall HML-2 transcript levels in ALS-derived and control bulk tissue samples, arguing against higher transcriptional activity of one or several HML-2 loci in ALS-derived tissue. Full-length Envelope protein was undetectable in protein lysates from ALS and control bulk tissue samples; smaller Env isoforms were seen although these did not obviously favor the disease. Our findings suggest that various HML-2-encoded proteins, some of them known to affect cell biology, may be expressed in ALS and control states in the central nervous system tissue types investigated in our study and thus may require further specific investigations. Our study furthermore calls for high-throughput strategies specifically suited for a comprehensive description of HML-2 transcription in order to better comprehend the role of HML-2 in ALS and potentially other neurodegenerative diseases. Such increased understanding is of special significance as the U.S. National Institutes of Health engages in a Phase I clinical trial investigating HERV-K(HML-2) suppression using antiretroviral therapy in volunteers with ALS (ClinicalTrials.gov Identifier: NCT02437110).

## Additional files

Additional file 1: **Table S1.** Tissue samples used in this study. **Table S2.** Relative cloning frequencies of *gag* amplicon-derived cDNAs from different HERV-K(HML-2) loci. (XLSX 29 kb)

Additional file 2: **Figure S1.** HERV-K(HML-2) locus-specific nucleotide differences in cDNA sequences derived from gag amplicon. **Figure S2.** HERV-K(HML-2) locus-specific nucleotide differences in cDNA sequences derived from an *env* amplicon. **Figure S3.** HERV-K(HML-2) locus-specific nucleotide differences in cDNA sequences derived from a *rec* amplicon. **Figure S4.** HERV-K(HML-2) locus-specific nucleotide differences in cDNA sequences derived from an np9 amplicon. **Figure S5.** Normalized levels of HERV-W transcripts identified in various ALS and control tissue samples. **Figure S6.** Normalized levels of HERV-W transcripts identified in various ALS and control tissue samples. **Figure S7.** Expression of HERV-K(HML-2) Env protein detected with the HERM-1811-5 antibody. **Figure S8.** Agarose gel photos of HML-2 *env*-specific endpoint RT-PCRs for subsequent HML-2 transcription profiling. **Figure S9.** RNA qualities and correlations with GAPDH C_t_-values. **Figure S10.** No correlation of relative HERV-K(HML-2) transcript levels with age or gender of donors. (PDF 2700 kb)

## Abbreviations

aa: Amino acid
ALS: Amyotrophic lateral sclerosis
bp: Base pairs
cDNA: Complementary DNA
Env: Envelope
GAPDH: Glyceraldehyde 3-phosphate dehydrogenase
H9-hESCs: H9 human embryonic embryonic stem cells
HEFs: Human embryonic fibroblasts
HERV: Human endogenous retrovirus
HML: Human MMTV-like
kDa: Kilodalton
LINE1/L1: Long interspersed element 1
LTR: Long terminal repeat
np9: Novel protein of 9 kDa
nt: Nucleotide
ORF: Open reading frame
PCR: Polymerase chain reaction
qPCR: Quantitative PCR
rec: Regulator of expression encoded by corf
RNA-Seq: RNA-sequencing
RT: Reverse transcription
RT-PCR: Reverse transcription PCR
SD: Standard deviation
SP: Signal peptide
SU/OM: Surface/outer membrane
TE: Transposable element
TM: Transmembrane
