# Supplementary material for: Transcriptional profiling of HERV-K(HML-2) in amyotrophic lateral sclerosis and potential implications for expression of HML-2 proteins
Source: Mol Neurodegener. 2018 Aug 2;13:39. doi: 10.1186/s13024-018-0275-3 (PMC6091006; doi:10.1186/s13024-018-0275-3)
Supplement: Supplementary file 2 — Figure S1. HERV-K(HML-2) locus-specific nucleotide differences in cDNA sequences derived from gag amplicon. Figure S2. HERV-K(HML-2) locus-specific nucleotide differences in cDNA sequences derived from an env amplicon. Figure S3. HERV-K(HML-2) locus-specific nucleotide differences in cDNA sequences derived from a rec amplicon. Figure S4. HERV-K(HML-2) locus-specific nucleotide differences in cDNA sequences derived from an np9 amplicon. Figure S5. Normalized levels of HERV-W transcripts identified in various ALS and control tissue samples. Figure S6. Normalized levels of HERV-W transcripts identified in various ALS and control tissue samples. Figure S7. Expression of HERV-K(HML-2) Env protein detected with the HERM-1811-5 antibody. Figure S8. Agarose gel photos of HML-2 env-specific endpoint RT-PCRs for subsequent HML-2 transcription profiling. Figure S9. RNA qualities and correlations with GAPDH Ct-values. Figure S10. No correlation of relative HERV-K(HML-2) transcript levels with age or gender of donors. (PDF 2700 kb) [file 13024_2018_275_MOESM2_ESM.pdf]

Transcriptional Profiling of HERV-K(HML-2) in Amyotrophic Lateral Sclerosis and Potential Implications for Expression of HML-2 Proteins

Jens Mayer[1*], Christian Harz [1], Laura Sanchez[2], Gavin C. Pereira[3], Esther Maldener[1], Sara R. Heras[2], Lyle W. Ostrow[4], John Ravits[5], Ron Batra[5], Eckart Meese[1], Jose Luis García-Pérez[2,6], John L. Goodier[3*].

1. Department of Human Genetics, University of Saarland, Homburg, Germany.
2. GENYO. Centre for Genomics and Oncological Research: Pfizer, University of Granada, Andalusian Regional Government, Granada, Spain.
3. McKusick-Nathans Institute for Genetic Medicine, Johns Hopkins University School of Medicine, Baltimore, MD USA.
4. Department of Neurology, Johns Hopkins University, Baltimore, MD 28217, USA.
5. Department of Neurosciences, School of Medicine, University of California at San Diego, USA.
6. MRC Human Genetics Unit, Institute of Genetics and Molecular Medicine (IGMM), University of Edinburgh, Western General Hospital, Edinburgh, UK.

Additional Information

Supplementary Fig. S1: HERV-K(HML-2) locus-specific nucleotide differences in cDNA sequences derived from *gag* amplicon.

Supplementary Fig. S2: HERV-K(HML-2) locus-specific nucleotide differences in cDNA sequences derived from an *env* amplicon.

Supplementary Fig. S3: HERV-K(HML-2) locus-specific nucleotide differences in cDNA sequences derived from a *rec* amplicon.

Supplementary Fig. S4: HERV-K(HML-2) locus-specific nucleotide differences in cDNA sequences derived from an *np9* amplicon.

Supplementary Fig. S5: Normalized levels of HERV-W transcripts identified in various ALS and control tissue samples.

Supplementary Fig. S6: Normalized levels of HERV-W transcripts identified in various ALS and control tissue samples.

Supplementary Fig. S7: Expression of HERV-K(HML-2) Env protein detected with the HERM-1811-5 antibody.

Supplementary Fig. S8: Agarose gel photos of HML-2 env-specific endpoint RT-PCRs for subsequent HML-2 transcription profiling.

Supplementary Fig. S9: RNA qualities and correlations with GAPDH C_t-values.

Supplementary Fig. S10: No correlation of relative HERV-K(HML-2) transcript levels with age or gender of donors.

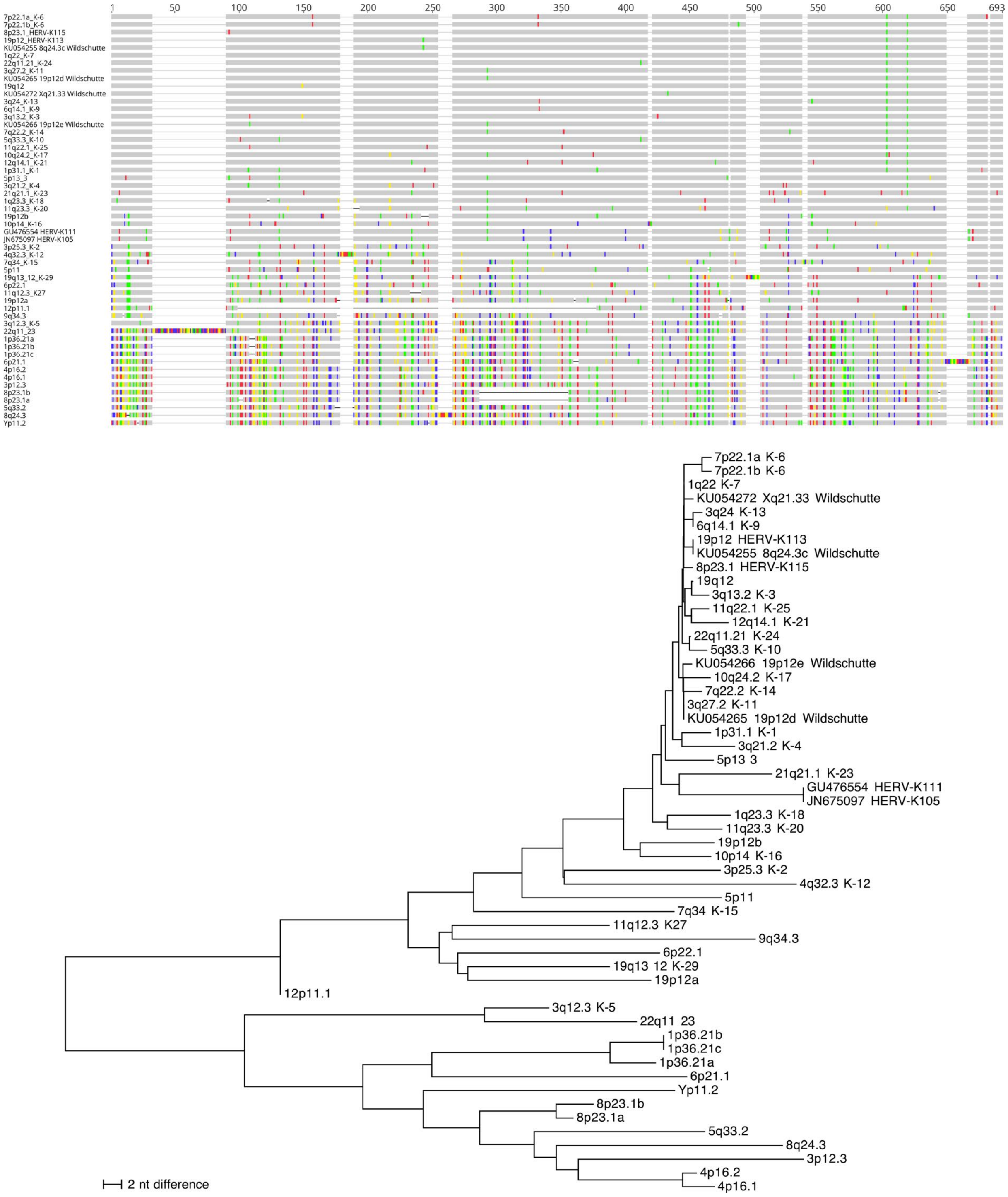

Supplementary Fig. S1: HERV-K(HML-2) locus-specific nucleotide differences for an RT-PCR amplicon located in *gag*. Shown are a multiple sequence alignment and Neighbour Joining tree depicting absolute numbers of nt differences of HERV-K(HML-2) loci within a *gag* amplicon region, including recently reported polymorphic, non-reference HML-2 proviral sequences. Positions with gaps were disregarded in pairwise comparisons for the tree building. Designations of particular HML-2 locus sequences are based on chromosomal bands and as used in the main paper text. See also Fig. 1 in the main paper.

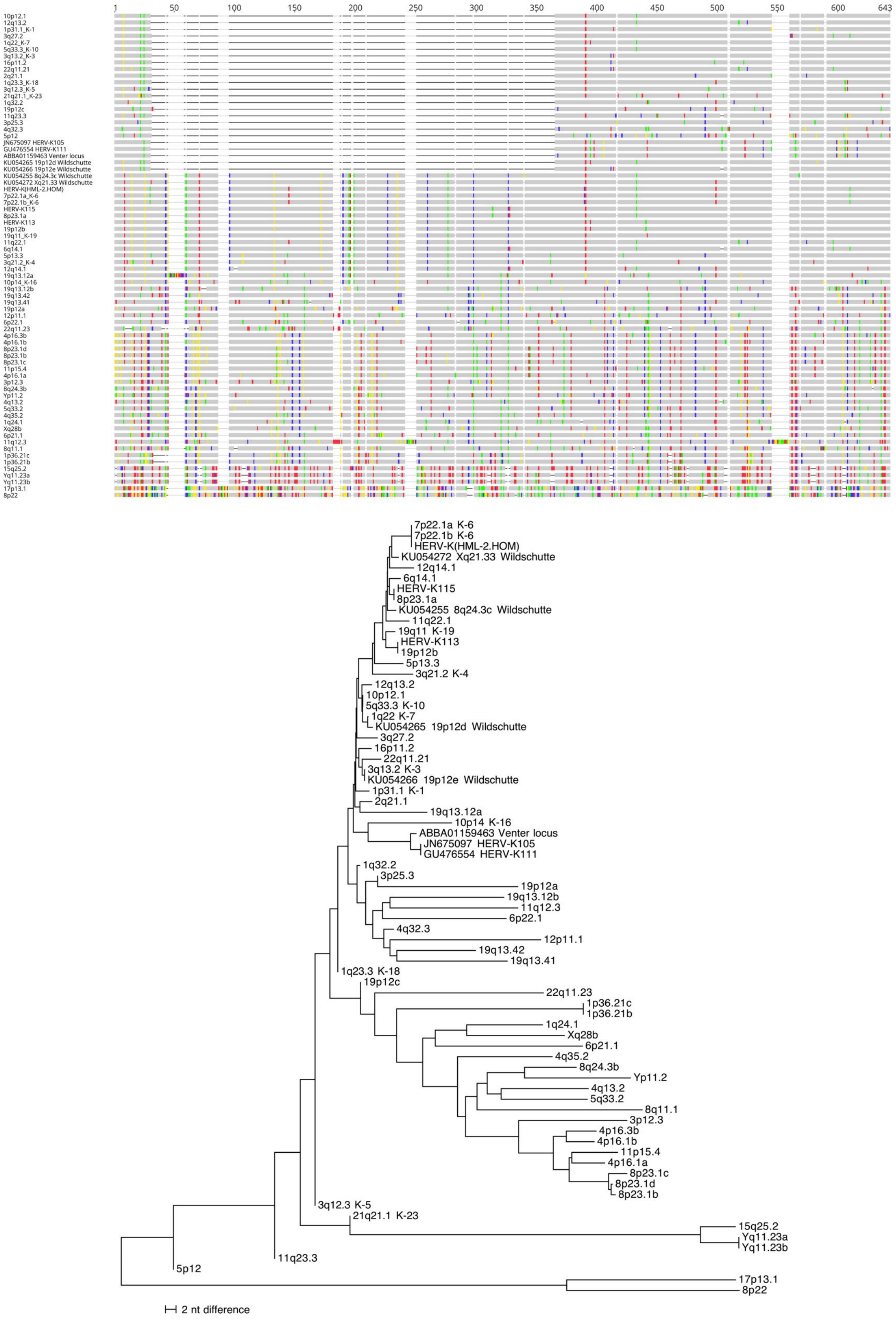

Supplementary Fig. S2: HERV-K(HML-2) locus-specific nucleotide differences for an RT-PCR amplicon located in *env*. Shown are a multiple sequence alignment and Neighbour Joining tree depicting absolute numbers of nt differences of HML-2 loci within *the* amplicon region located in the *env* gene's 5' region. See Fig. S1 for more details.

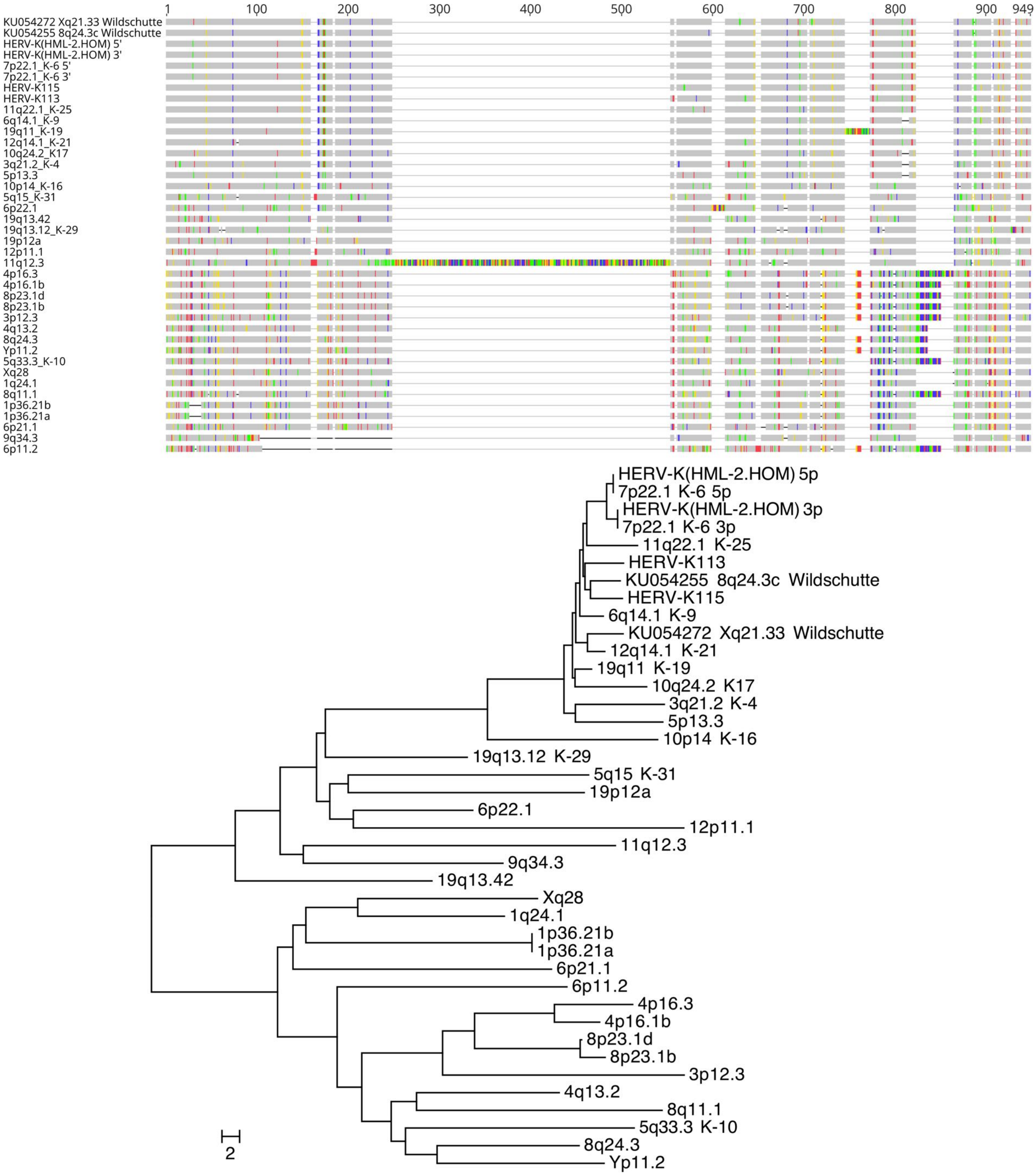

Supplementary Fig. S3: HERV-K(HML-2) locus-specific nucleotide differences for a RT-PCR amplicon targeting spliced *rec* transcript. Absolute numbers of sequence differences are depicted for an RT-PCR region amplified from *rec* exons 2 and 3 (see Fig. 1 in the main paper) and excluding primer binding regions. See Fig. S1 and S2 for more details.

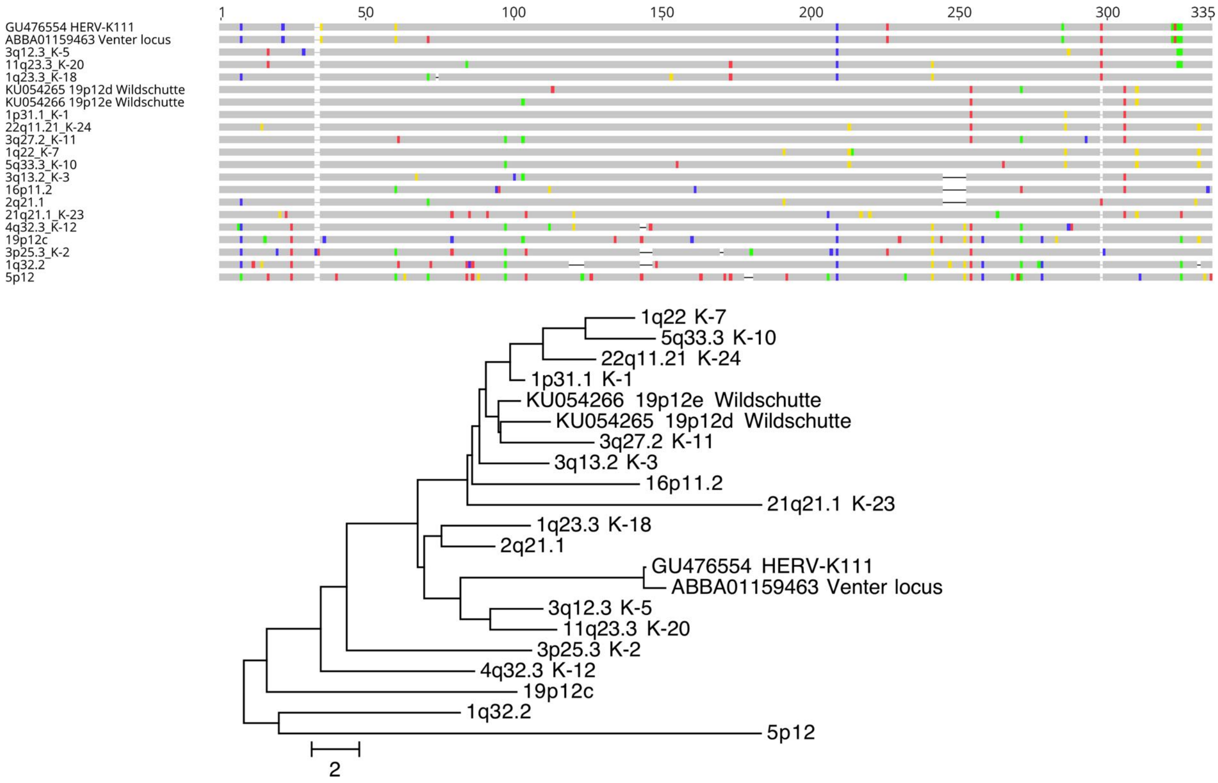

Supplementary Fig. S4: HERV-K(HML-2) locus-specific nucleotide differences for a RT-PCR amplicon targeting spliced *np9* transcript. Absolute numbers of sequence differences are depicted for an RT-PCR region amplified from *np9* exons 2 and 3. See Fig. S1-S3 for more details.

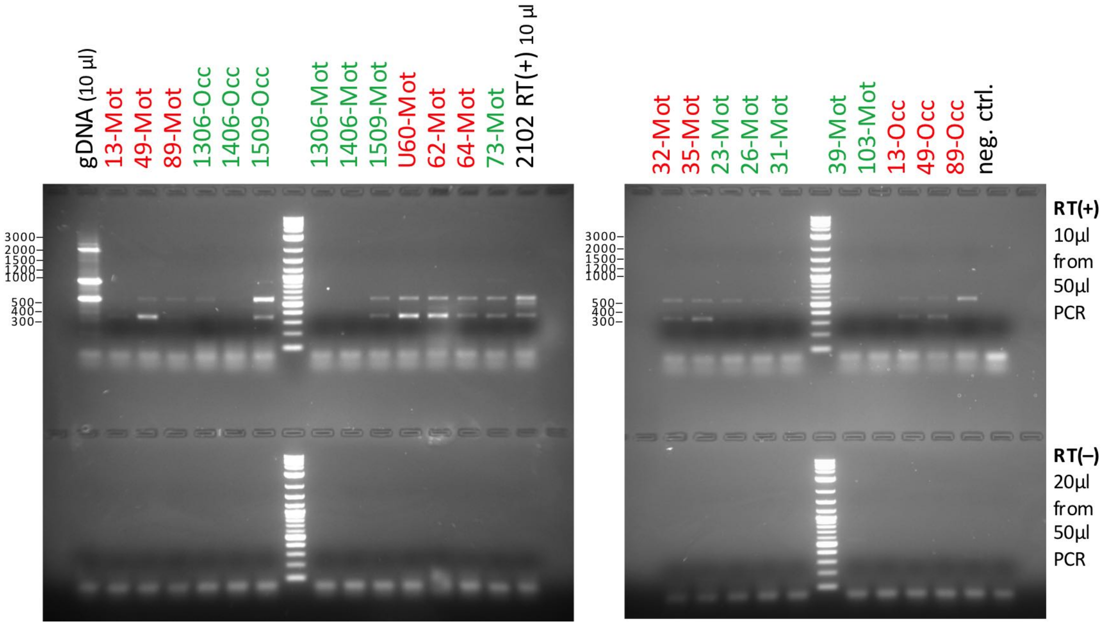

Supplementary Fig. S5: Results from RT-PCRs amplifying portions of *rec* and *np9* transcripts from various ALS-derived and control samples. Samples derived from ALS patients and controls are labelled in red and green, respectively. For PCRs of RT(+) reactions, 10 µl out of 50 µl PCR volumes were electrophoresed in 1.5% TAE agarose gels each. For better verification of absence of contaminating gDNA in RT reactions, 20 µl out of 50 µl RT(–) reactions were electrophoresed. Reaction volumes loaded from control reactions (gDNA, 2102Ep cells) are indicated. RT-PCR products of approximately 580 bp and 360 bp represent *rec* and *np9* transcripts, respectively. PCR of gDNA also generated two products around ~2.1 kb (the larger one clearly fainter) from full-length type 1 and type 2 HML-2 *env* genes. Note that relative amounts of *rec* and *np9* RT-PCR products are variable between some samples. See the main paper text for more details.

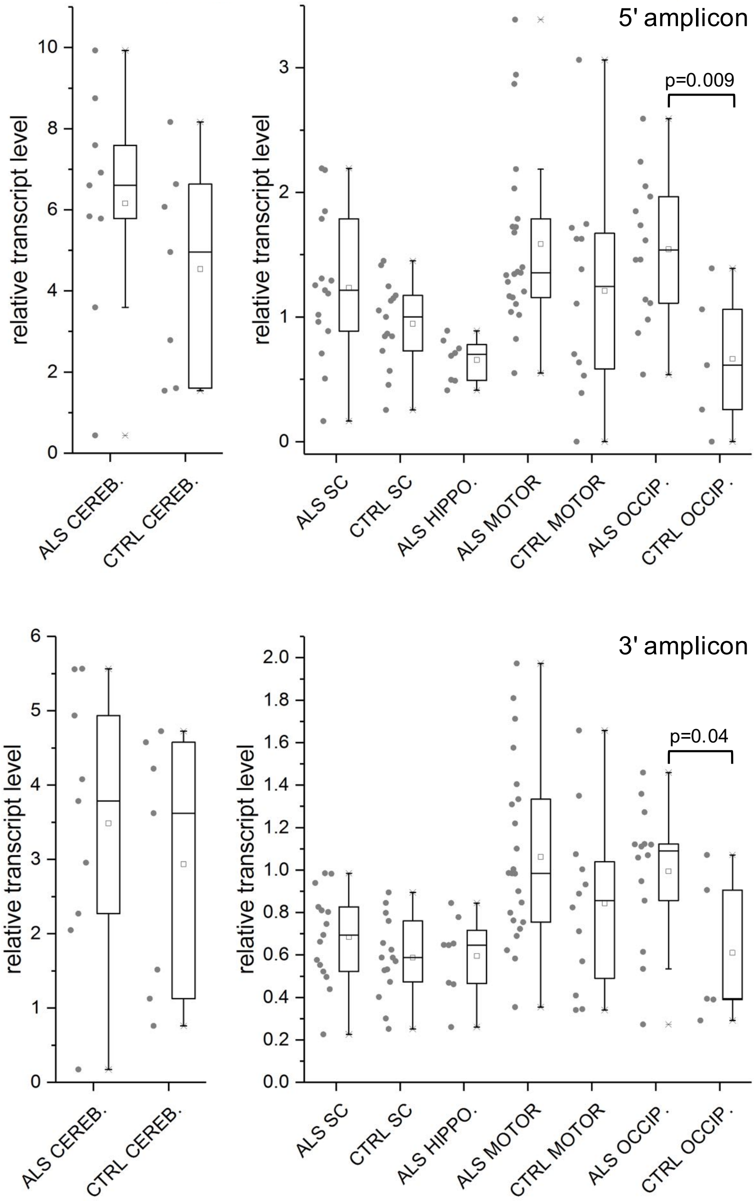

Supplementary Fig. S6: Boxplots of normalized levels of HERV-W transcripts identified in various ALS and control tissue samples. Transcript levels were determined following a previously established RT-qPCR strategy and utilizing two different amplicons within the HERV-W *env* gene region (Schmitt et al. 2013; J Virol 87:13837-52). For both the HERV-W *env* 5' and 3' amplicons only transcript levels in tissue samples from occipital region were statistically significantly different when comparing ALS patients with controls. Respective p-values from a Student's t-test are given. Transcript levels from Motor neuron samples were compared by a Wilcoxon-signed-rank test because normal distribution could not be confirmed for transcript levels of ALS motor neuron samples (Shapiro-Wilk test, $p<0.05$).

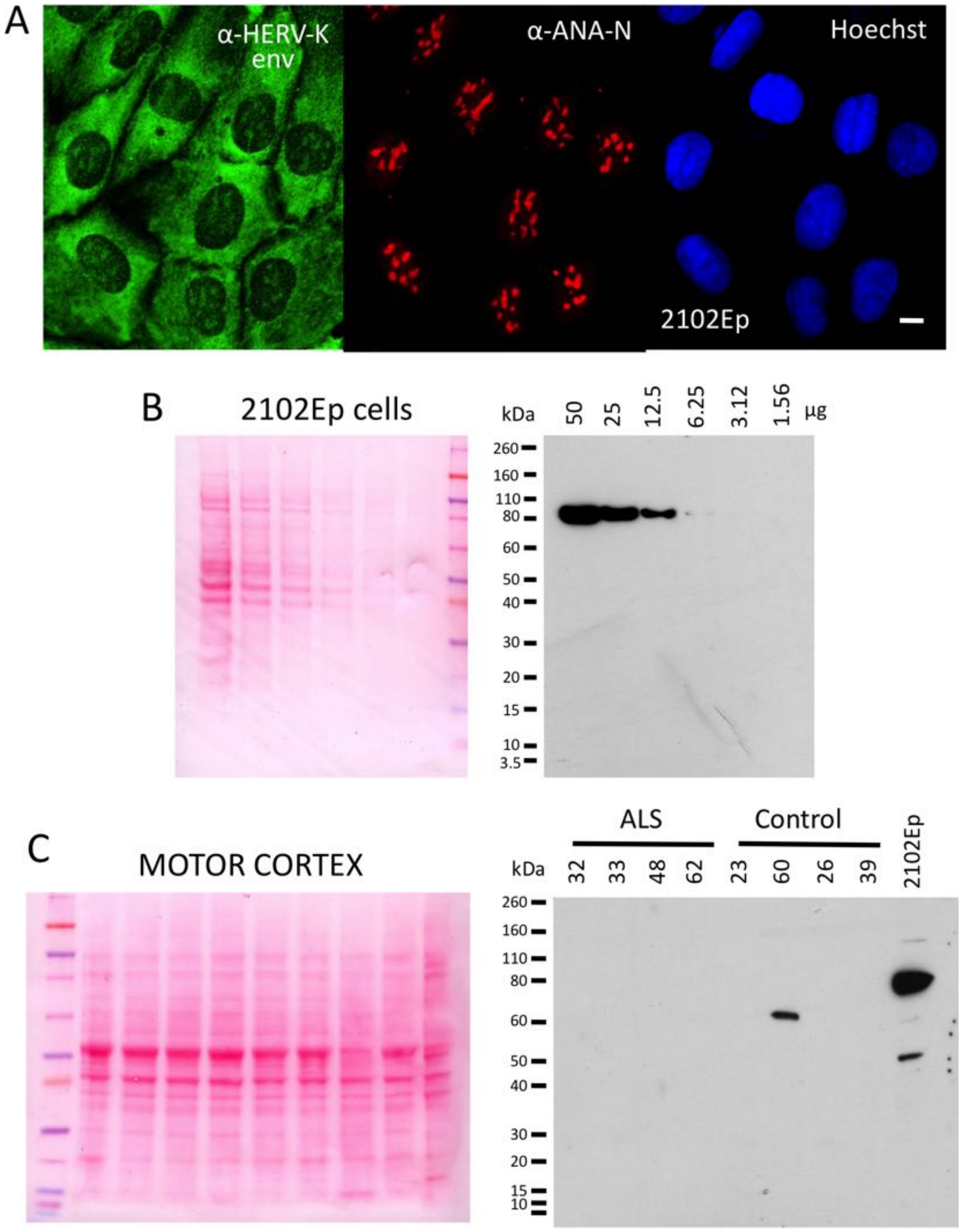

Supplementary Fig. S7 (see next page for legend)

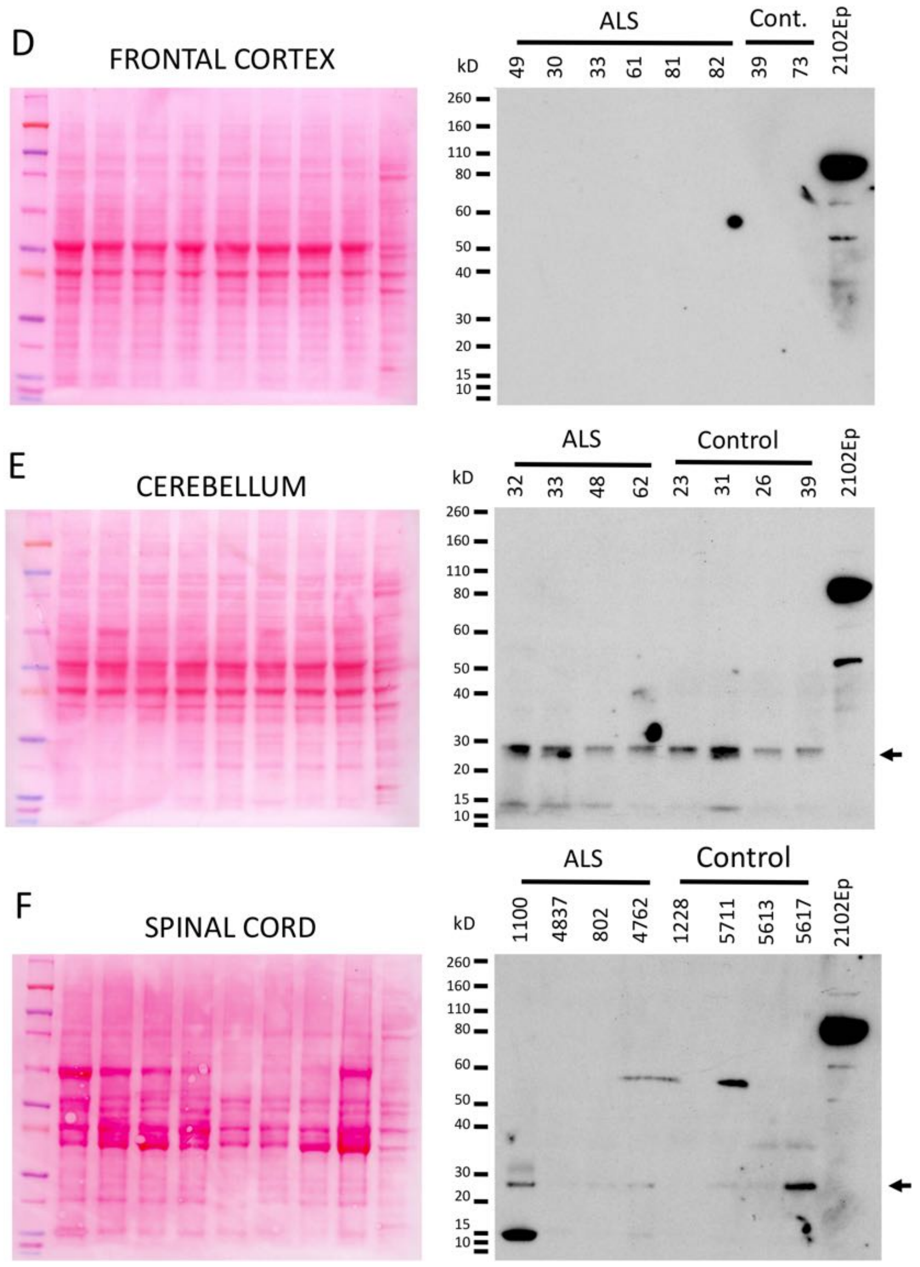

Supplementary Fig. S7: Expression of HERV-K(HML-2) Env protein detected with the HERM-1811-5 antibody (Austral Biologicals). **A**) Strong detection of HML-2 Env protein in fixed 2102Ep cells by immunofluorescence (left panel). Cells were also co-stained with α-ANA-N human auto-immune sera that cross-reacts with nucleolar proteins (middle panel), and Hoechst to mark nuclei (right panel). The size bar is 10 µm. **B**) Dilution series of 2102Ep whole cell lysate showing that the α-HML-2 Env antibody can detect the ~80 kDa full-length Env in as little as 6 µg of lysate. (**C-F**) Western blotting detection of HML-2 Env protein in normal and ALS-associated motor cortex (**C**), frontal cortex (**D**), cerebellum (**E**) and spinal cord (**F**) tissue lysates (right-hand panels). Following Western blotting, PVDF transfer membranes were stained with Ponceau S to confirm equal loading of proteins (left-hand panels). 2102Ep cell lysate was loaded in the right-most lane of each gel as a control for expression of full-length Env protein. Fifty µg of whole cell lysates were loaded in each lane. Nine µl of Novex Sharp Pre-stained Protein Standard was loaded, shown in the left-most lane of the PonceauS-stained gels. The putative 27 kDa unglycosylated HML-2 Env-TM protein is marked by arrows in panels (**E**) and (**F**).

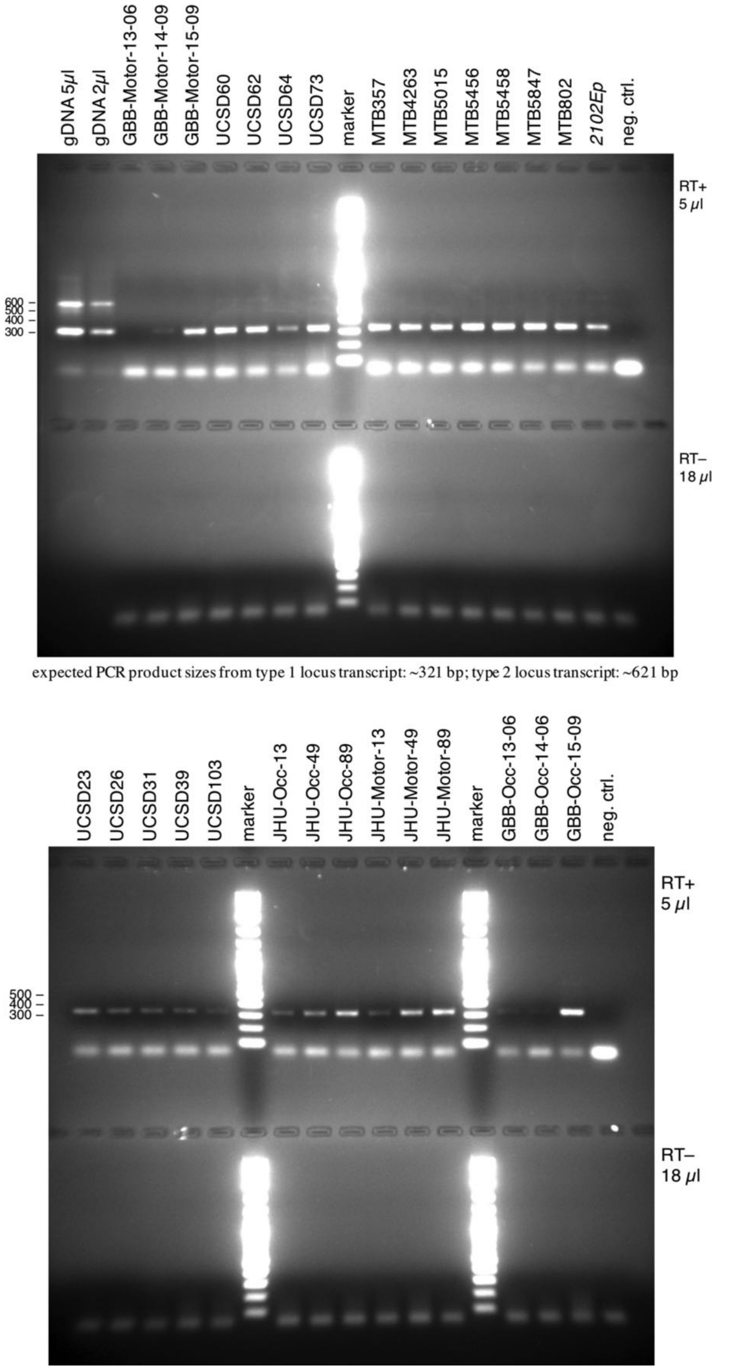

Supplementary Fig. S8: Agarose gel photos of HML-2 *env*-specific endpoint RT-PCRs for subsequent HML-2 transcription profiling. Larger volumes of RT-PCR reactions from RT(–) controls, compared to RT(+), were loaded onto gels as indicated. A positive control PCR was run using genomic DNA (gDNA) as template, with 5 µl and 2 µl from a 50 µl PCR reaction loaded onto the agarose gel. Type 1 and type 2 PCR amplicons, ~321 bp and ~621 bp, respectively, are visible. Note that, despite efficient amplification of a type 2 amplicon from gDNA, RT-PCR products representing type 1 *env* transripts were predominantly amplified; products representing type 2 transcripts appear faint even when the gel image is overexposed.

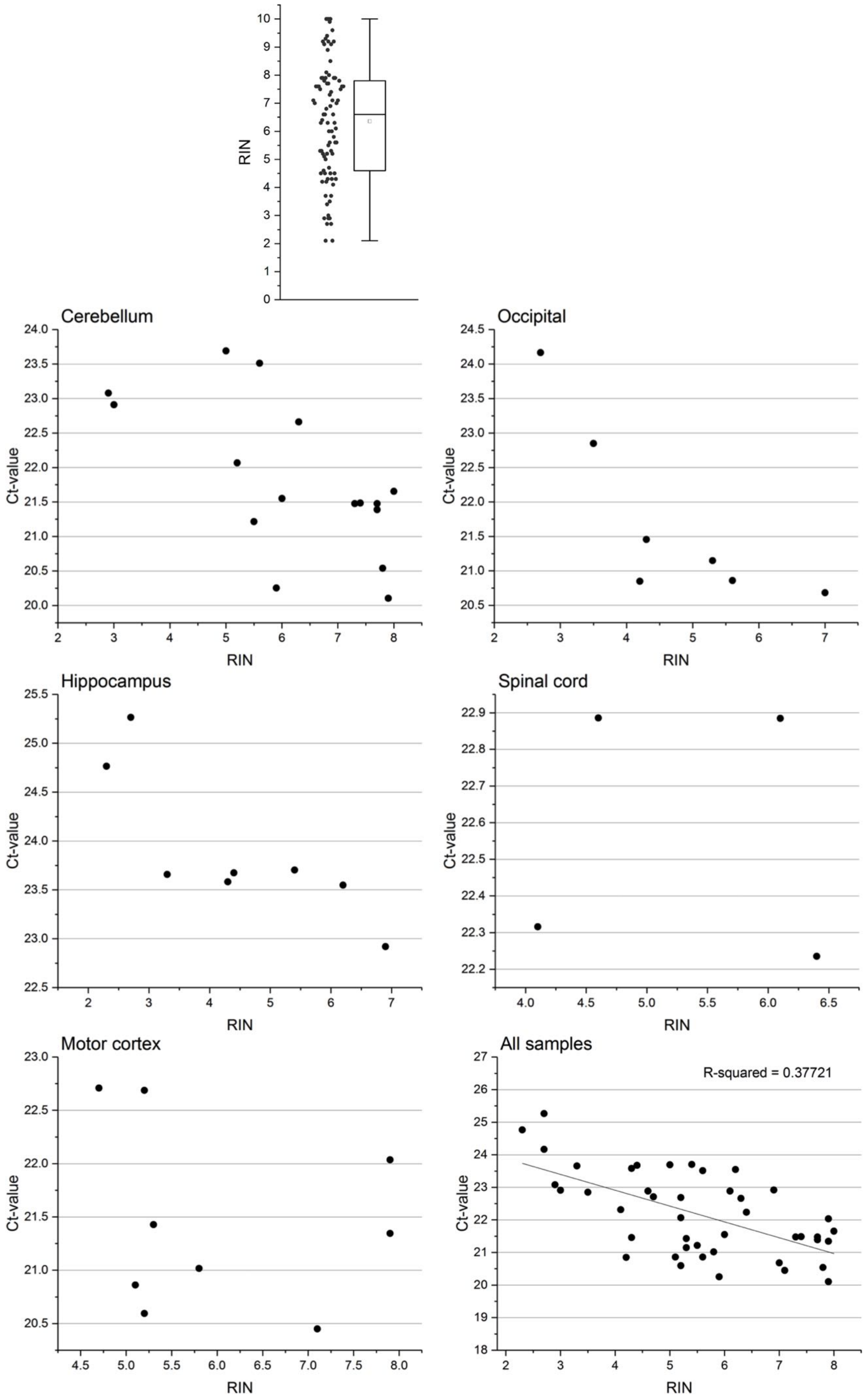

Supplementary Fig. S9: RNA qualities and correlations with GAPDH C_t-values. The top boxplot depicts RNA Integrity Numbers (RINs) of RNAs used in this study. The scatter plots below depict, separate for each tissue type, correlations of RIN and C_t-values of measured GAPDH transcript levels for RNAs used for HERV-K(HML-2) expression analysis. The bottom-right scatter plot combines all RIN and C_t-values. No effects of RNA quality on Ct-values were noted when regarding each tissue type separately. A minor effect was seen only when combining all RNAs.

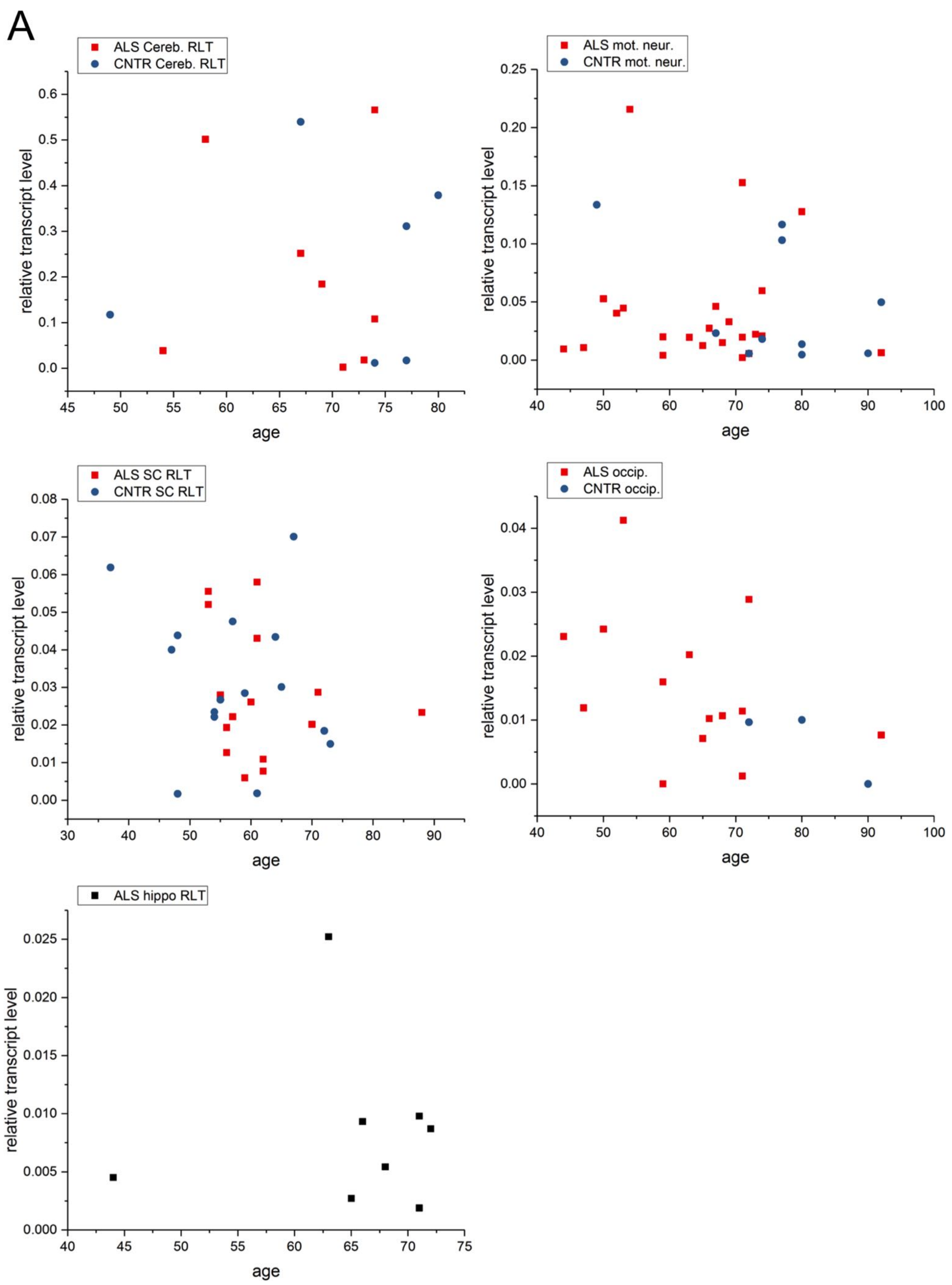

Supplementary Fig. S10 (see next page for legend)

B

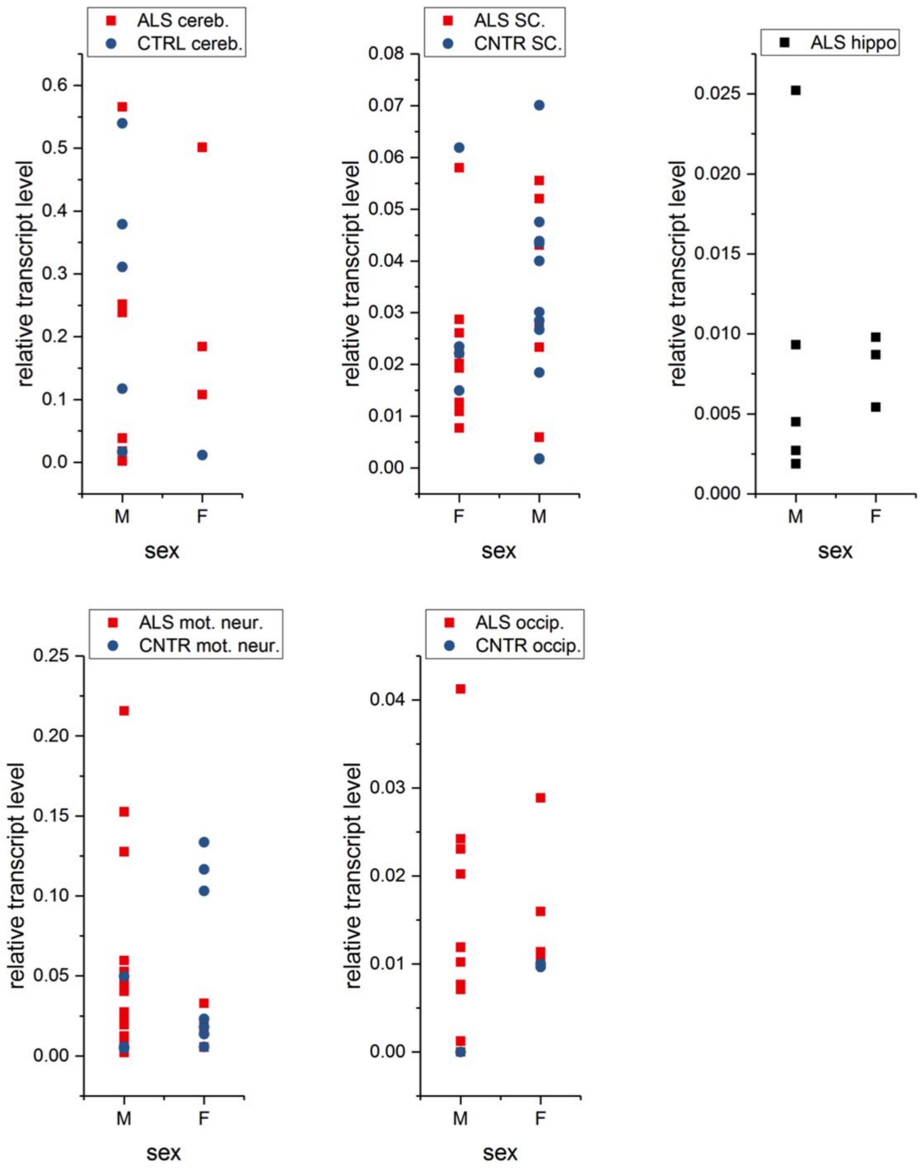

Supplementary Fig. S10: No correlation of relative HERV-K(HML-2) transcript levels with age or gender of donors. Scatter plots depicting correlations between (**A**) age of donors and (**B**) gender of donors and HERV-K(HML-2) transcript levels as measured by RT-qPCR (see the main paper text for details). Correlations are given separate for each tissue type. ALS and control samples are indicated by red squares and blue dots, respectively. Hippocampus samples were all from ALS.
